# Multilineage Differentiation Potential of Human Dental Pulp Stem Cells—Impact of 3D and Hypoxic Environment on Osteogenesis In Vitro

**DOI:** 10.3390/ijms21176172

**Published:** 2020-08-26

**Authors:** Anna Labedz-Maslowska, Natalia Bryniarska, Andrzej Kubiak, Tomasz Kaczmarzyk, Malgorzata Sekula-Stryjewska, Sylwia Noga, Dariusz Boruczkowski, Zbigniew Madeja, Ewa Zuba-Surma

**Affiliations:** 1 Department of Cell Biology, Faculty of Biochemistry, Biophysics and Biotechnology, Jagiellonian University, 30-387 Krakow, Poland; anna.labedz-maslowska@uj.edu.pl (A.L.-M.); natalia.bryniarska@outlook.com (N.B.); andrzej.kubiak@ifj.edu.pl (A.K.); sylwia.noga@uj.edu.pl (S.N.); z.madeja@uj.edu.pl (Z.M.); 2 Department of Experimental Neuroendocrinology, Maj Institute of Pharmacology, Polish Academy of Sciences, 31-343 Krakow, Poland; 3 Institute of Nuclear Physics, Polish Academy of Sciences, 31-342 Krakow, Poland; 4 Department of Oral Surgery, Faculty of Medicine, Jagiellonian University Medical College, 31-155 Krakow, Poland; tomasz.kaczmarzyk@uj.edu.pl; 5 Laboratory of Stem Cell Biotechnology, Malopolska Centre of Biotechnology, Jagiellonian University, 30-387 Krakow, Poland; malgorzata.sekula@uj.edu.pl; 6 Polish Stem Cell Bank, 00-867 Warsaw, Poland; dariusz.boruczkowski@pbkm.pl

**Keywords:** stem cells, dental pulp stem cells, osteogenesis, biomaterials, tissue engineering, regenerative medicine

## Abstract

Human dental pulp harbours unique stem cell population exhibiting mesenchymal stem/stromal cell (MSC) characteristics. This study aimed to analyse the differentiation potential and other essential functional and morphological features of dental pulp stem cells (DPSCs) in comparison with Wharton’s jelly-derived MSCs from the umbilical cord (UC-MSCs), and to evaluate the osteogenic differentiation of DPSCs in 3D culture with a hypoxic microenvironment resembling the stem cell niche. Human DPSCs as well as UC-MSCs were isolated from primary human tissues and were subjected to a series of experiments. We established a multiantigenic profile of DPSCs with CD45^−^/CD14^−^/CD34^−^/CD29^+^/CD44^+^/CD73^+^/CD90^+^/CD105^+^/Stro-1^+^/HLA-DR^−^ (using flow cytometry) and confirmed their tri-lineage osteogenic, chondrogenic, and adipogenic differentiation potential (using qRT-PCR and histochemical staining) in comparison with the UC-MSCs. The results also demonstrated the potency of DPSCs to differentiate into osteoblasts in vitro. Moreover, we showed that the DPSCs exhibit limited cardiomyogenic and endothelial differentiation potential. Decreased proliferation and metabolic activity as well as increased osteogenic differentiation of DPSCs in vitro, attributed to 3D cell encapsulation and low oxygen concentration, were also observed. DPSCs exhibiting elevated osteogenic potential may serve as potential candidates for a cell-based product for advanced therapy, particularly for bone repair. Novel tissue engineering approaches combining DPSCs, 3D biomaterial scaffolds, and other stimulating chemical factors may represent innovative strategies for pro-regenerative therapies.

## 1. Introduction

The mesenchymal stem/stromal cells (MSCs) represent an adult stem cell (SCs) population that exhibits tri-lineage differentiation potential [1]. The MSCs can be isolated from various tissues, including bone marrow (BM), umbilical cord blood and Wharton’s jelly of the umbilical cord (UC), adipose tissue, peripheral blood, synovium or dental pulp [2]. For distinguishing better between MSCs of various origins, the International Society for Cellular Therapy (ISCT) proposed minimal criteria for defining MSCs in 2006 that included the following: (i) an ability to adhere to plastic surfaces under standard culture conditions in vitro; (ii) a specific antigenic profile including at least 95% positivity for CD105, CD73 and CD90 antigen expression with parallel negativity (less than 2%) for CD45, CD34, CD14 or CD11b, CD79α or CD19 and HLA-DR), and (iii) a capacity to differentiate into the cells of mesodermal lineages, such as osteoblasts, chondroblasts and adipocytes in vitro [3]. Moreover, MSCs derived from various tissues may cross the boundaries of the germ layers and differentiate into cardiomyocyte- or endothelial-like cells (with parallel ability to form capillary-like structures on Matrigel) upon exposure to lineage-specific growth factor cocktails [4,5,6,7]. Recent evidence indicates that murine BM-MSCs may differentiate into hepatocytes, lung epithelial cells, myofibroblasts, and renal tubular cells in vivo [8]. Thus, based on their wide differentiation potential and their pro-regenerative potential in injured tissues, MSCs are promising “candidates” as medicinal products employed in in vivo applications for advanced therapy in humans. MSCs have been widely evaluated in preclinical studies as well as with a multitude of clinical trials focused on several tissue injuries, including bone defects [9] and other defects such as cartilage injury [10]. Of note, recent evidence demonstrates the biological variability (including differentiation capacity) within the MSCs of various origins [11]. Thus, the selection of the appropriate source of MSCs might be critical for the effective regeneration of injured tissues following their application in vivo.

BM-derived MSCs represent the gold standard for MSCs used in research and clinical applications (predominantly in autologous transplantations) in recent years [12]. However, the application of autologous MSCs may have some limitations. BM-derived MSCs isolated from elderly patients exhibit decreased biological activity, including osteogenic potential [13], which may result in limited outcomes of the treatment with these cells. Moreover, MSCs isolated from the tissues of patients suffering from chronic conditions like diabetes or systemic lupus erythematosus may also exhibit altered biological properties impairing their pro-reparative functions in autologous applications [14,15]. Nowadays, growing evidence indicates that umbilical cord tissue represents an attractive alternative source of MSCs for allogenic applications, which eliminates the limitations of autologous MSCs as well as the need for the invasive procedure of tissue harvesting that is required for BM [16,17]. Moreover, UC tissue possesses higher accessibility and lower ethical concerns in comparison with BM. Importantly, UC-MSCs have been demonstrated to exhibit similar transcriptomic profiles with respect to the expression of stemness- and bone development-related genes to BM-MSCs (harvested from healthy donors), demonstrating the great promise for umbilical cord tissue as an alternative source of MSCs [17].

In 2000, Gronthos et al. described a unique population of SCs residing in human dental pulp referred to as dental pulp stem cells (DPSCs). They exhibit mesenchymal characteristics, including adhesion to plastic surfaces, fibroblast-like morphology, lack of expression of CD14, CD34, and CD45, as well as the potential to differentiate into osteoblasts [18]. Moreover, DPSCs show antigenic profile similar to BM-MSCs, along with greater proliferation capacity when compared to that of BM-MSCs [18]. Although DPSCs predominantly originate from the neural crest, which is an ectodermal structure, they are considered as MSCs or MSC-like cells [19,20,21]. Thus, the unique developmental origin of DPSCs may indicate their ability to produce neural cells and their impact on neural tissue regeneration, conferring certain advantages in comparison with other sources of MSCs [22,23,24,25,26]. DPSCs exhibit capability not only to differentiate into neural-like cells [25] but also to secret factors enhancing endogenous repair mechanisms [27]. The unique features of DPSCs resulted in the first clinical application for the treatment of neurodegenerative diseases [28]. Moreover, DPSCs, when cultured under appropriate conditions, are known to exhibit osteogenic potential [18,29]. While long bones develop from mesoderm during the process of endochondral ossification, flat bones of the skull, similar to the mandible, are formed during the intramembranous ossification from ectomesenchymal cells derived from neural crest SCs [30,31]. Once DPSCs were identified as MSCs derived from the neural crest, they were recognized as a potential promising candidate for the repair of defects of the jaws [20]. Interestingly, DPSCs may also be used in the innovative strategies for dentin regeneration [32].

Following numerous clinical trials employing MSCs of various origins, some concerns about their efficacy and safety have been raised [33]. In many cases, results of clinical applications were less favourable than expected, with the observation of certain side effects upon the administration of MSCs into a suboptimal niche [33]. One of the reasons for the limited pro-regenerative capacity of MSCs may be the lack of well-established protocols for MSC isolation, expansion, and preparation of a cell therapy product for in vivo applications [33] as well as the use of an inappropriate cell source or differentiation strategy [32]. Importantly, the standard culture conditions with plastic or glass surfaces (representing two-dimensional culture, 2D) do not fully mimic the three-dimensional (3D) microarchitecture of SC niches [34]. In this context, the use of novel 3D scaffolds (created by f.e. hydrogels) for cells ex vivo propagation, differentiation, or as a carrier for the administration of cells, provides a new exciting approach to enhance the biological potential of MSCs for the repair of the injured tissue. MSCs encapsulated in alginate beads exhibit greater osteogenic differentiation potential than MSCs in traditional cell culture, which was confirmed by the higher expression of mRNAs corresponding to osteoblast-specific genes (e.g., *Runx2, Col1A1*, sclerostin or dental matrix protein-1) [35]. Similar observations have been described for DPSCs differentiated in scaffolds, such as Matrigel or collagen-based sponges, in vitro [36].

Oxygen (O_2_) concentration in the culture environment represents another important factor that should be taken into consideration during the differentiation of MSCs both in vitro and in vivo. It has been established that the normoxic environment does not reflect the oxygen concentration in SC niches [37]. Importantly, in numerous reports, MSCs were shown to proliferate faster when cultured in hypoxic conditions with 1% [38], 2% [39], or 3% O_2_ [40], compared to that in the normoxic atmosphere. In contrast, a decrease in the proliferation of rat BM-MSCs in the presence of 1% O_2_ was reported owing to the upregulation of p27 protein correlating with the downregulation of cyclin D expression [41]. Hsu et al. demonstrated that the ex vivo culture of human MSCs in the hypoxic (1% O_2_) environment decreases their osteogenic differentiation. The hypoxic environment at the site of cell transplantation in the injured tissues may be a critical factor impairing the efficacy of MSCs to differentiate following their transplantation [42], which, along with the spatial niche organization, provides a unique environment for functional behaviour of the cells in vivo.

Thus, this study aimed to examine the differentiation potential of ectoderm-derived human DPSCs in vitro, in comparison with other human MSC fractions, such as the Wharton’s jelly-derived MSCs from the umbilical cord (UC-MSCs). We also evaluated osteogenic differentiation of human DPSCs in 3D cultures and hypoxic microenvironment in vitro as well as examined their selected functional properties, including proliferation and metabolic activity.

## 2. Results

### 2.1. Human Dental Pulp Harbours a Population of Adherent Cells with MSC Characteristics

**Figure 1:**
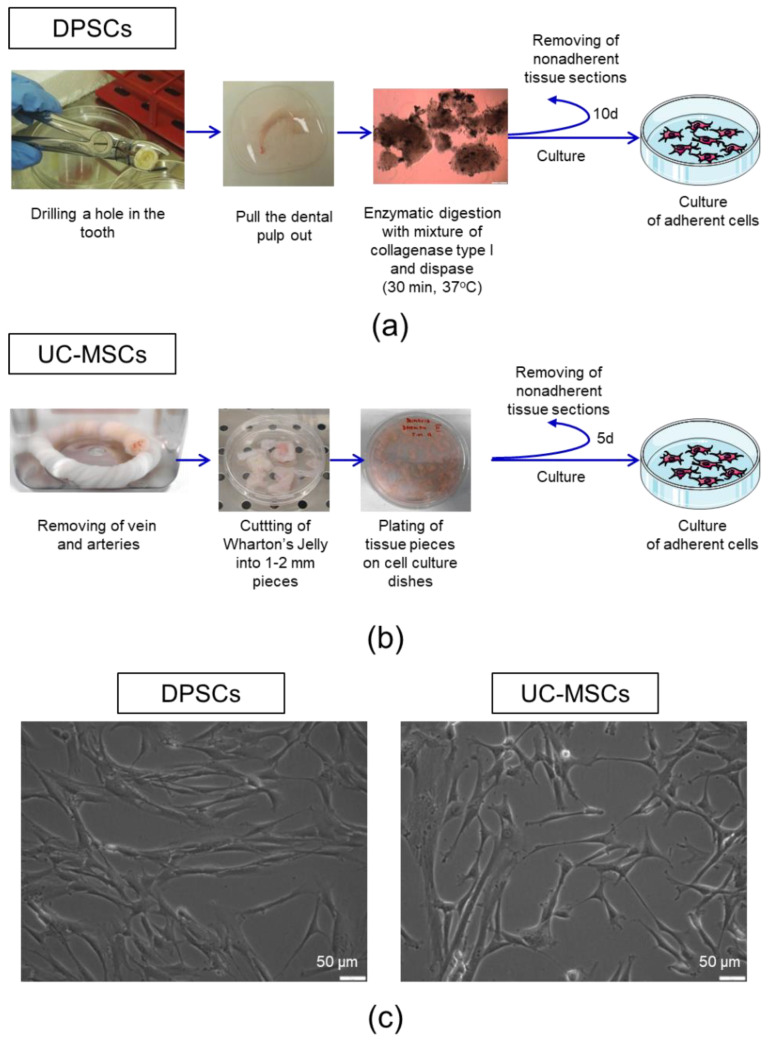
Isolation procedure and morphology of dental pulp stem cells (DPSCs) and umbilical cord Wharton’s jelly-derived mesenchymal stem/stromal cells (UC-MSCs). (**a**) Isolation of DPSCs from pulp tissue. The upper part of the tooth was drilled and the dental pulp was extracted. The dental pulp was enzymatically digested by a mixture of collagenase I and dispase. The isolated cells and tissue sections were seeded onto cell culture plates in a complete cell culture medium. On day 10 post-seeding, non-adherent cells and tissue pieces were removed. (**b**) Isolation of UC-MSCs from Wharton’s jelly. The umbilical cord was washed with PBS to remove residual cord blood, and arteries and vein were further dissected. Wharton’s Jelly tissue was cut into 12 mm pieces and placed on the tissue culture dishes in a complete cell culture medium. On day five post-seeding, non-adherent cells and tissue pieces were removed. (**c**) Representative images of the morphology of DPSCs (left) and UC-MSCs (right). Scale bars: 50 µm.

Accumulating evidence indicates that the dental pulp contains a unique population of adherent cells with mesenchymal characteristics, including adhesion to plastic surfaces, fibroblast-like morphology, lack of expression of CD14, CD34 or CD45 and potential to differentiate into osteoblasts in vitro [18,43]. Thus, in the current study, we developed and optimized the protocol for the isolation of such cells with the corresponding characteristics of MSCs (Figure 1a). The dental pulp extracted from the permanent teeth of healthy human donors was subjected to enzymatic digestion to isolate a mixture of different populations of cells, which were further seeded on cell culture plates. After 10 days, we obtained a population of adherent cells that were proliferated further. Moreover, we also isolated UC-MSCs from human UC Wharton’s Jelly tissue (Figure 1b) by employing a similar approach, which were used as a “classic” control MSCs for comparison with the dental pulp- derived cells in vitro.

We observed that both DPSCs, as well as UC-MSCs, demonstrated adhesion to plastic surfaces when maintained under standard culture conditions in vitro. Dental pulp-derived cells exhibit a morphology of spindled-shaped, fibroblast-like cells, similar to UC-MSCs (Figure 1c). By using a flow cytometry platform, we analysed the antigenic profile of isolated cells following the minimal criteria for defining multipotent mesenchymal stem/stromal cells published by the ISCT [3].

**Figure 2:**
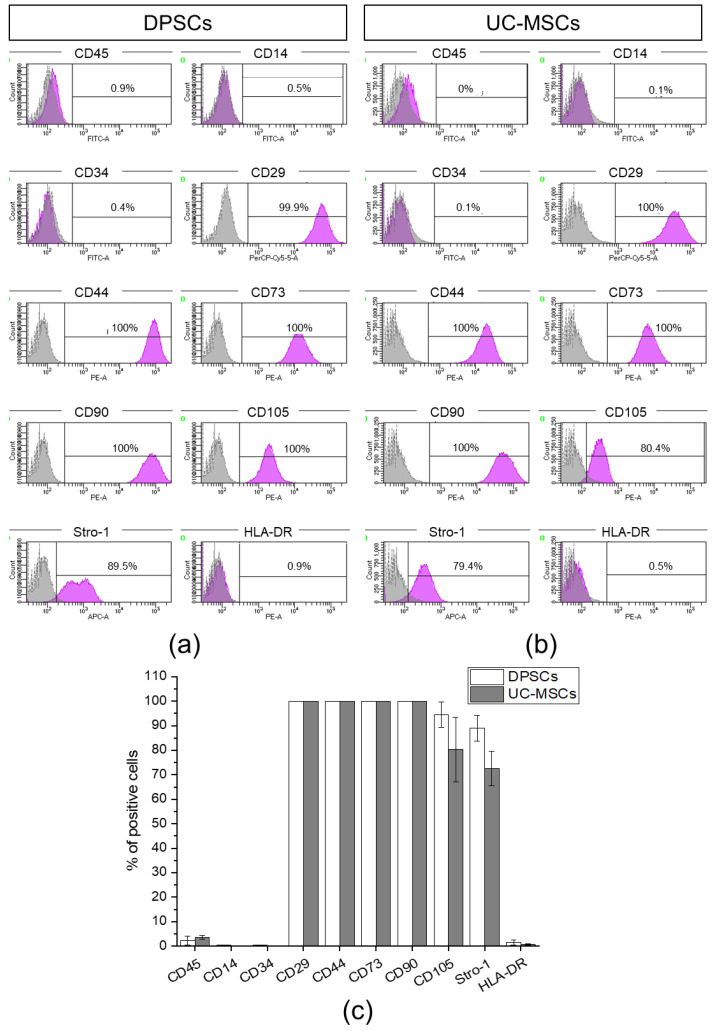
Antigenic profile of DPSCs and UC-MSCs with flow cytometry. Expression of MSC-negative markers (D45, CD14, CD34), MSC- positive markers (CD29, CD44, CD73, CD90, CD105, Stro-1), and HLA-DR antigen on DPSCs and UC-MSCs. (**a**) Representative histograms of the expression of analysed antigens on DPSCs. (**b**) Representative histograms of the expression of analysed antigens on UC-MSCs. The peaks of unstained cells (grey) were overlaid with the peak for analysed antigen (violet). (**c**) Quantitative data representing the percentage content of DPSCs or UC-MSCs positive for analysed antigens. Results are presented as mean ± SD, *n* = 3.

We demonstrated that the population of dental pulp-derived cells isolated in this study exhibited a high expression of MSC-specific markers, such as CD29, CD44, CD73, CD90, CD105 and Stro-1, and does not express markers specific to hematopoietic cells, such as CD45, CD14, CD34 or HLA-DR antigen (Figure 2a). UC-MSCs also express antigens typical for MSCs, such as CD29, CD44, CD73, CD90 and Stro-1, and, in parallel, do not possess CD45, CD14, CD34 and HLA-DR antigens on their surface that are considered markers of hematopoietic cells (Figure 2b). Thus, the multiantigenic phenotype of the dental pulp-derived cells is similar to the antigenic profile of UC-MSCs as shown in Figure 2c. Thus, based on their ability to adhere to plastic surfaces and antigenic profiles, we confirmed the identity of the isolated dental pulp-derived cells as previously described, representing the subpopulation of MSCs.

### 2.2. DPSCs Exhibit Wide Differentiation Potential In Vitro

**Figure 3:**
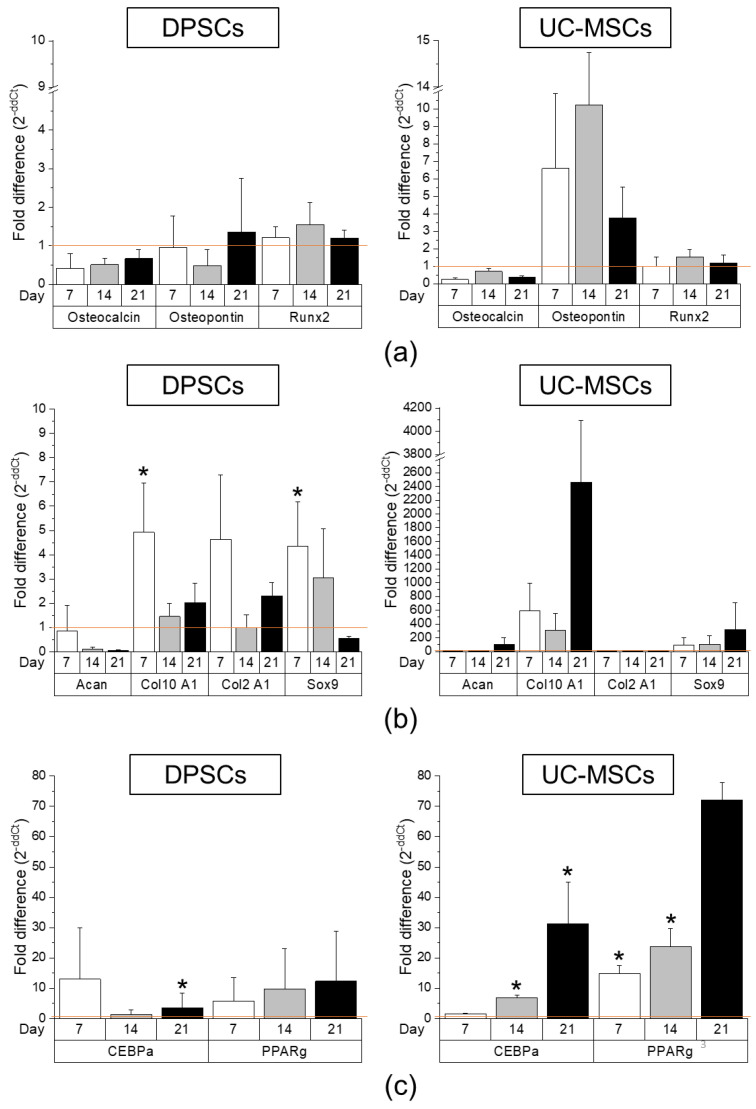
Comparison of tri-lineage differentiation potential of DPSCs and UC-MSCs by real-time RT-PCR. (**a**) Quantitative analysis of mRNA expression for osteogenesis related genes (osteocalcin, osteopontin, *Runx2*) in DPSCs (left) and UC-MSCs (right). (**b**) Quantitative analysis of mRNA expression for chondrogenesis related genes (*Acan, Col10A1, Col2A1, Sox9*) in DPSCs (left) and UC-MSCs (right). (**c**) Quantitative analysis of mRNA expression for adipogenesis related genes (*CEBPα, PPARγ*) in DPSCs (left) and UC-MSCs (right). Cells were cultured in a StemPro osteogenesis differentiation kit, StemPro chondrogenesis differentiation kit, and StemPro adipogenesis differentiation kit for 7, 14, and 21 days, respectively. Fold differences in expression (2^−ddCt^) of analysed genes in control cells cultured in standard cell culture medium (undifferentiated) were calculated as 1.0 and marked by a solid line. Graphs present different scales. Results are presented as mean ± standard error of the mean (SEM), *n* = 3 (every sample prepared for each DPSCs line derived from each donor were run in duplicates); *t*-test, (*) *p* < 0.05 vs. undifferentiated cells.

**Figure 4:**
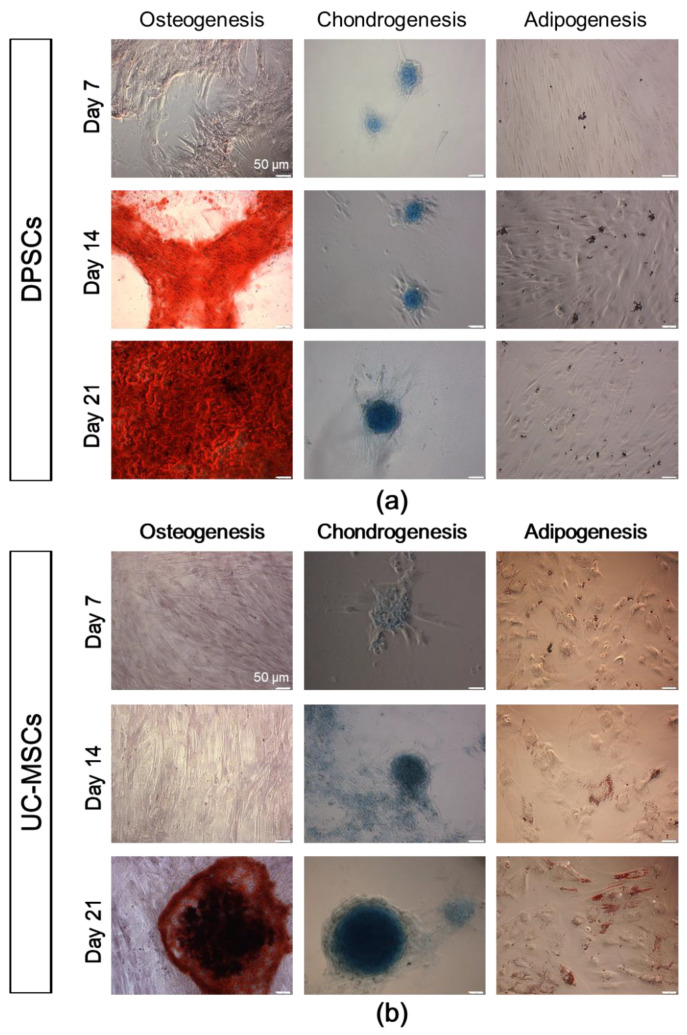
Tri-lineage differentiation potential of DPSCs and UC-MSCs in an in vitro culture demonstrated by histochemical staining. (**a**) Representative images of DPSCs differentiated into osteoblasts, chondroblasts and adipocytes. (**b**) Representative images of UC-MSCs differentiated into osteoblasts, chondroblasts, and adipocytes. DPSCs and UC-MSCs were cultured in a StemPro osteogenesis differentiation kit, StemPro chondrogenesis differentiation kit, or StemPro adipogenesis differentiation kit. On days 7, 14, and 21 of differentiation, DPSCs and UC-MSCs were fixed with paraformaldehyde and stained with Alizarin Red S (red staining of calcium phosphate deposits that are a characteristic of osteogenic differentiation), Alcian Blue (blue staining of sulphated proteoglycans that are a characteristic of chondrogenic differentiation) or Oil Red O (brownish red oil droplets that are a characteristic of adipogenic differentiation). Scale bars: 50 µm.

In the next step, to answer the question about the biological potential of DPSCs with respect to their pro-regenerative ability in injured tissues, we first analysed the tri-lineage differentiation potential of such cells compared to UC-MSCs in vitro. For that purpose, the DPSCs and UC-MSCs were differentiated into osteoblasts, chondroblasts, and adipocytes after 7, 14 and 21 days in tissue-specific differentiation media. We observed that both DPSCs and UC-MSCs exhibit tri-lineage differentiation potential (as shown in Figure 3 and Figure 4, respectively), which also confirmed their MSC phenotype as defined by minimal criteria recommended by ISCT [3].

In the case of osteogenic differentiation, we analysed the expression of osteogenesis-related genes during the differentiation process of both MSC populations, such as Runx2, osteocalcin and osteopontin, in comparison with the control (undifferentiated) cells, which were cultured under standard culture conditions. We observed that the expression levels of transcription factor Runx2 and osteocalcin (a marker of bone formation) were comparable between DPSCs and UC-MSCs, whereas the fold change in expression of osteopontin (a protein expressed in maturated bone tissue) was elevated in UC-MSCs, notably on the 14th-day post-stimulation (Figure 3a, Table S1). Real-time RT-PCR results obtained for both MSC populations were compared with those of the control (undifferentiated) cells cultured in a standard cell culture medium (mRNA levels in such cells were calculated as 1.0).

The histochemical staining of cells differentiated into osteoblasts demonstrated larger deposits of calcium phosphate (indicated by red-coloured deposits of calcium phosphate) that were observed following DPSC differentiation when compared to the differentiation of UC-MSCs. Moreover, the deposits were observed earlier (at 14 days) in the case of DPSC osteogenic differentiation compared to those with differentiation of UC-MSCs (Figure 4). The comparable expression of the genes between DPSCs and UC-MSCs along with the higher formation of calcium phosphate deposits following DPSC differentiation may demonstrate a higher osteogenic differentiation potential of the DPSCs compared to that of the UC-MSCs.

The DPSCs, as well as UC-MSCs, were successfully differentiated into chondroblasts in vitro (Figure 3b and Figure 4, respectively). In the case of DPSCs, we observed increased expression of *Sox9* transcription factor mRNA on days 7 and 14 of differentiation, compared to that in the undifferentiated cells, which confirmed their chondrogenic differentiation potential. However, the expression of *Sox9* gene was higher in UC-MSCs in comparison with DPSCs. We did not observe any significant change in the expression of *Col2A1* between both types of cells, while the fold change in the expression of *Col10A1* was higher in the UC-MSCs compared to that in the DPSCs (Figure 3b, Table S2). Recent evidence indicates that *Col10A1* is a marker of hypertrophic chondrocytes, which may be implicated as the principal factor driving bone growth. It has also been observed in skeletal dysplasia and osteoarthritis disorders [44]. The histochemical staining of DPSCs and UC-MSCs that were differentiated into chondroblasts indicated extracellular secretion of sulphated proteoglycans (indicated by blue coloured staining) by both types of MSCs, and the kinetics of differentiation seemed to be similar between both populations of SCs (Figure 4).

When focusing on adipogenic differentiation, the mRNA expression corresponding to *PPARγ* adipogenesis-related transcription factor on day 21 as well as *CEBPα* protein expression on day 14 was significantly higher in the UC-MSCs in comparison with that in the DPSCs (Figure 3c, Table S3). Moreover, the presence of oil droplets indicating an ongoing process of adipogenesis was typically observed in both cell fractions on the 14th day of differentiation. The results considering the level of demonstrate higher adipogenic differentiation of UC-MSCs compared to DPSCs.

Taken together, our first analyses confirmed that DPSCs may be successfully differentiated into osteoblasts, chondroblasts, and adipocytes similar to other “classic” MSC populations such as UC-MSCs. The histochemical analyses of the final cell phenotypes confirmed a higher ability of DPSCs to differentiate into osteoblasts compared to UC-MSCs, which are primarily restricted to chondrogenic and adipogenic differentiation.

**Figure 5:**
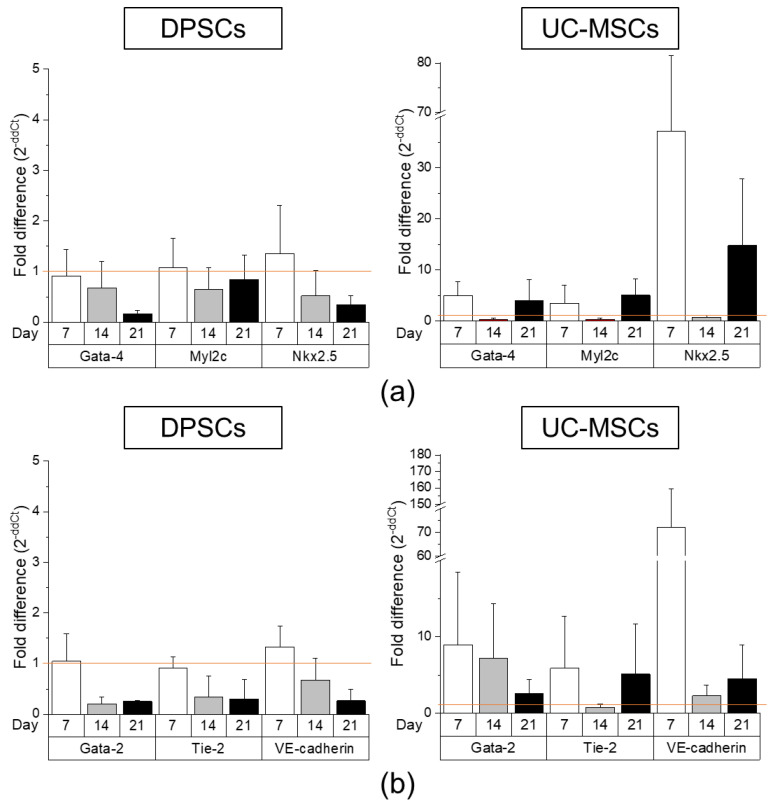
Comparison of cardiomyogenic and endothelial differentiation potential of DPSCs and UC-MSCs by Real-Time PCR. (**a**) Quantitative analysis of mRNA expression of cardiomyogenesis related genes (*Gata-4, Nkx2.5, Myl2c*) in DPSCs (left) and UC-MSCs (right). Cells were cultured in DMEM/F12 supplemented with 2% FBS and 10 ng/mL basic fibroblast growth factor (bFGF), 10 ng/mL vascular endothelial growth factor (VEGF) and 10 ng/mL transforming growth factor β1 (TGF-β1) for 7, 14 and 21 days. (**b**) Quantitative analysis of mRNA expression for endothelial related genes (*Gata-2, Tie-2*, VE-cadherin) in DPSCs (left) and UC-MSCs (right). Cells were cultured in EGM-2MV endothelial cell growth medium for 7, 14 and 21 days. Fold differences in the expression (2^−ddCt^) of analysed genes in control cells cultured in standard cell culture medium (undifferentiated) were calculated as 1.0 and marked by a solid line. Graphs present different scales. Results are presented as mean ± SEM, *n* = 3 (every sample prepared for each DPSCs line from each donor was run in duplicate); *t*-test, *p* < 0.05 vs. undifferentiated cells.

**Figure 6:**
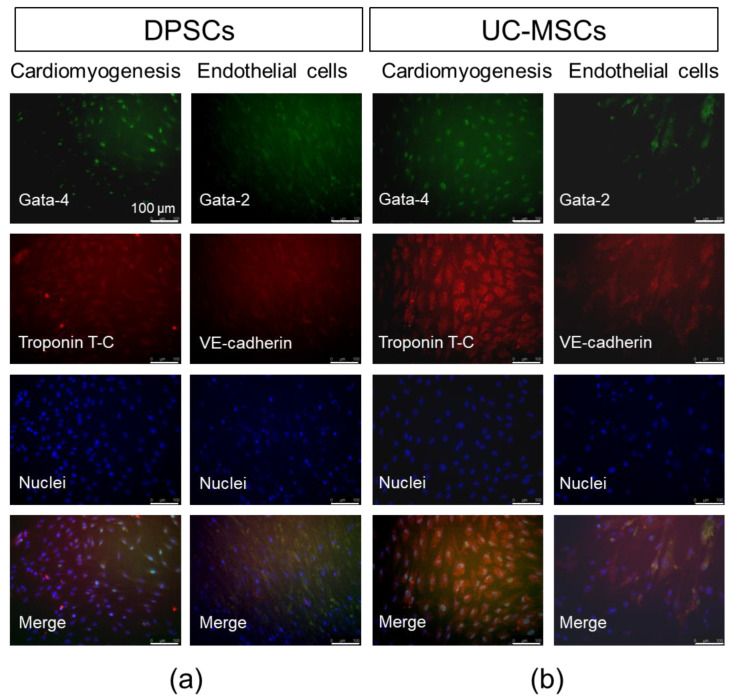
Cardiomyogenic and endothelial differentiation of DPSCs and UC-MSCs in vitro on day 7. (**a**) Representative images of cardiomyogenic and endothelial marker expression in DPSCs. (**b**) Representative images of cardiomyogenic and endothelial marker expression in UC-MSCs. In the case of cardiomyogenic differentiation, cells were cultured in DMEM/F12 supplemented with 2% FBS and 10 ng/mL bFGF, 10 ng/mL VEGF and 10 ng/mL TGF-β1. On day 7, cells were fixed, permeabilized, and stained against intranuclear transcription factor *Gata-4* (Alexa Fluor 488, green) and troponin T-C (Alexa Fluor 546, red), whereas nuclei were co-stained with DAPI (blue). In the case of endothelial differentiation, cells were cultured in EGM-2MV. On day 7, cells were fixed, permeabilized, and stained against intranuclear transcription factor *Gata-2* (Alexa Fluor 488, green) and VE-cadherin (Alexa Fluor 546, red), whereas nuclei were co-stained with DAPI (blue). Cells were analysed with Leica DMI6000B ver. AF7000 fluorescent microscope. Scale bars: 100 μm.

To establish whether DPSCs exhibit any cardiomyogenic potential in vitro, they were cultured in cardiomyogenesis stimulating medium as previously described [6]. We analysed mRNA expression of cardiac markers such as *Gata-4, Nkx2.5* and *Myl2c* after 7, 14 and 21 days following the induction of differentiation. We observed that the expression of these genes was markedly elevated after 7 and 21 days of cardiomyogenic differentiation induction in UC-MSCs than that in DPSCs (Figure 5a and Table S4). Moreover, both types of MSCs express intranuclear cardiac transcription factor *Gata-4* as well as cytoplasmic structural protein troponin T-C after seven days of differentiation (Figure 6). This may suggest that DPSCs possess the lower capacity for cardiac cell phenotypes, when culture under lineage-specific conditions, in comparison with other MSC fractions, such as UC-MSCs.

To assess the potential angiogenic differentiation capacity of DPSCs, in comparison with UC-MSCs, we launched a long-term culture of these cells in proangiogenic medium containing VEGF (EGM-2MV). The angiogenic potential was examined both at the mRNA and protein levels. The highest expression of angiogenesis-related genes (*Gata-2, Tie2*, VE-cadherin) was observed in UC-MSCs on day 7 of differentiation, whereas the DPSCs were unresponsive to proangiogenic stimulation (expression of the proangiogenic genes was at the same level as in unstimulated DPSCs; Figure 5b, Table S5). Interestingly, the enhanced expression of these genes was also observed in UC-MSCs on the 14th and 21st days of proangiogenic stimulation. However, in the immunocytochemical staining, we did not observe any prominent expression of proangiogenic transcription factor Gata-2 or cell membrane protein VE-cadherin supporting angiogenesis (Figure 6).

Collectively, we observed the following features of DPSCs as cells that (i) exhibit higher osteogenic differentiation capacity, (ii) demonstrate comparable chondrogenic and adipogenic differentiation potential, and (iii) possess limited ability for cardiac or endothelial phenotype, in comparison with other “classic” MSCs (UC-MSCs).

### 2.3. 3D Encapsulated DPSCs Exhibit Higher Differentiation Capacity into Osteoblasts in Vitro

As shown previously, the DPSCs exhibit a higher osteogenic potential compared to the UC-MSCs. Based on the fact that osteogenic differentiation leading to bone formation is a process that takes place in the regular in vivo 3D niche of developing organism [45], we encapsulated the DPSCs in a hydrogel matrix to mimic such 3D niche in vitro and further analysed their morphology, proliferation, metabolic activity, and osteogenic potential in both normoxic or hypoxic culture environment.

**Figure 7:**
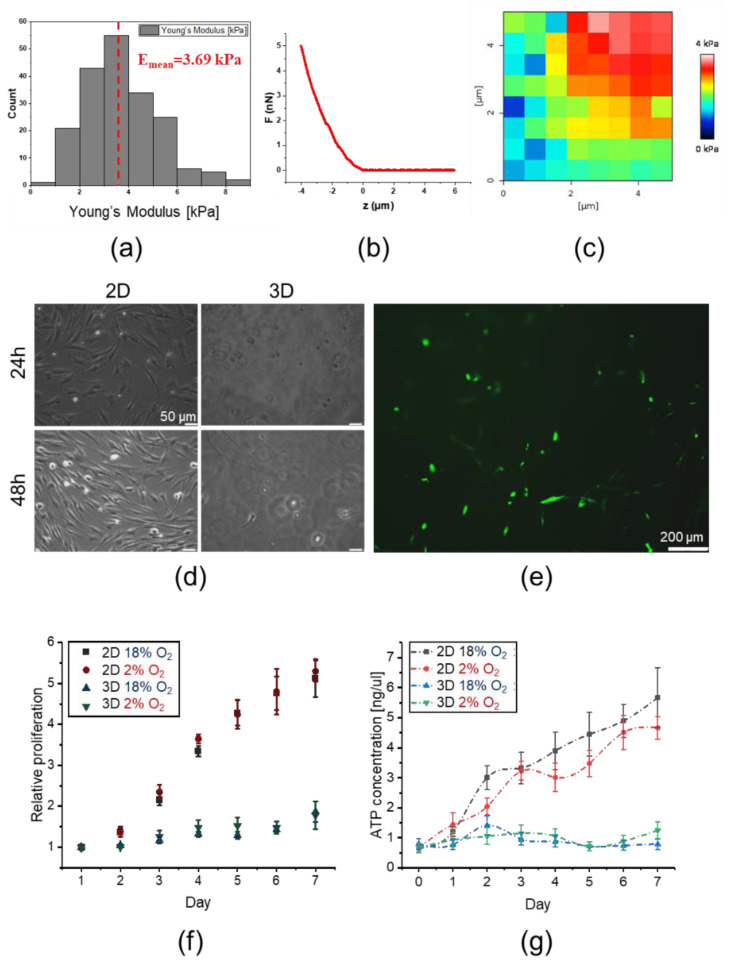
Mechanical properties of the hydrogel matrix and their impact on morphology, proliferation, and metabolic activity of DPSCs. (**a**) Young’s modulus distributions of hydrogel matrix by AFM. (**b**) Exemplary force curve recorded on the hydrogel. (**c**) Exemplary elasticity map of peptide hydrogel. (**d**) Morphology of DPSCs encapsulated in hydrogel (3D) or seeded on the surfaces coated with gelatin (2D) at 24 and 48 h post-seeding. DPSCs were cultured in DMEM/F12 supplemented with 10% FBS and cultured under standard culture conditions (5% CO_2_, normoxia). (**e**) Morphology of DPSCs encapsulated in hydrogel (3D culture) on day 4 post-seeding. Cells were stained with fluorescein diacetate and analysed with Leica DMI6000B ver. AF7000 fluorescent microscope to visualize their morphology. (**f**) The proliferation of DPSCs in 2D and 3D cultures in the environment containing about 18% or 2% O_2_ analysed every 24 h until day 7. The analyses were conducted using the Cell Counting Kit-8. (**g**) Metabolic activity of DPSCs in 3D and 2D culture in the environment containing about 18% or 2% O_2_ measured every 24 h until day 7. The analyses were conducted using the ATP Lite Luminescence assay kit. The results from proliferation and metabolic activity are presented as mean ± SEM, *n* = 3 (every sample prepared for each DPSCs line from each donor was analysed in triplicate).

Young’s moduli of hydrogel matrix measured using AFM were normally distributed (assessed by the Shapiro–Wilk test) with a mean value of E = 3.69 ± 1.49 kPa (Figure 7a). Elasticity maps demonstrate a heterogeneous distribution of elastic modulus (Figure 7b,c), thus providing more realistic conditions for cell growth. The resulting Young’s modulus was in the range of physiological tissue elasticity (~1–100 kPa, [34]) demonstrating rather highly deformable substrate properties. For proliferating cells encapsulated inside hydrogels, such gel does not constitute a barrier. Cells may be able to generate protrusive forces during cellular divisions and can be released into the surrounding environment [46].

We further observed that the DPSCs encapsulated in the 3D hydrogel exhibited a round shape 24 h post mixing, whereas certain flattened cells exhibiting a spindle-shaped morphology were observed after 48 h, indicating a phenotypical change. In contrast, DPSCs seeded on cell culture plates (2D) exclusively exhibited spindle-shaped morphology at both time points as expected in standard 2D conditions (Figure 7d,e). On the first day of 2D and 3D cultures, the relative proliferation of DPSCs in an environment containing 2% or approximately 18% of O_2_ was the same (Figure 7f). Between days 2 and 7 after culture initiation, we observed a greater relative proliferation of DPSCs in 2D culture compared to that in the 3D culture. However, we did not observe any influence of O_2_ concentration on the proliferation ratio of DPSCs in both 2D and 3D culture conditions at fixed-time intervals (Figure 7f). The increased proliferation of DPSCs in 2D culture was correlated with the higher metabolic activity of these cells (Figure 7g). DPSCs in 3D culture exhibited lower metabolic activity along with their lower proliferation. We also did not observe any significant impact of O_2_ concentration on the levels of ATP production in DPSCs in 2D and 3D cell culture (Figure 7g).

**Figure 8:**
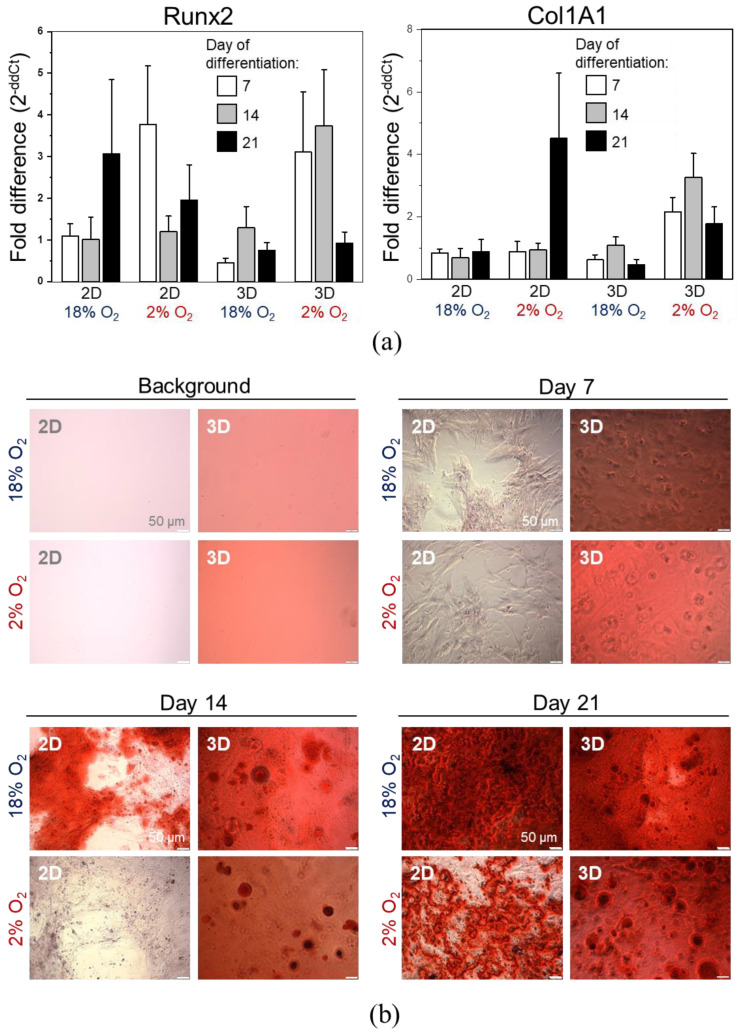
Osteogenic differentiation of DPSCs encapsulated in hydrogel (3D) or seeded on the surface coated with gelatin (2D) cultured in hypoxic (2% O_2_) or normoxic (about 18% O_2_) environment. (**a**) Quantitative analysis of mRNA expression for osteogenesis associated genes (*Col1A, Runx2*) in DPSCs on days 7, 14, and 21 of differentiation. Fold change in the expression of analysed genes in control cells before differentiation was calculated as 1.0 and marked by a solid line. Results are presented as mean ± SEM, *n* = 3 (every sample was analysed in duplicate). *p* < 0.05 (*t*-test). (**b**) Representative images of DPSCs differentiated into osteoblasts on days 7, 14, and 21 days of differentiation. Panel “Background” contains the representative images of 2D and 3D surfaces (standards plastic dish and hydrogel without cells, respectively) stained with Alizarin Red S solution to visualize background staining (on day 7 post surface preparation). Panels “Day 7”, “Day 14”, “Day 21” demonstrate representative images of DPSCs cultured in StemPro osteogenesis differentiation kit. On days 7, 14 and 21, DPSCs were fixed with paraformaldehyde and stained with Alizarin Red S (red staining of deposits of calcium phosphate is a characteristic for osteogenic differentiation).

In the next step, we conducted in vitro osteogenic differentiation of DPSCs in 2D or 3D culture in the presence of 2% or approximately 18% of O_2_. After seven days of stimulation, the concentration of mRNA for Runx2 was elevated by three times in DPSCs cultured in both 2D and 3D conditions in the presence of hypoxia (2% O_2_) in comparison with undifferentiated cells. The elevated expression of Runx2 in 3D culture in an environment containing 2% O_2_ was sustained up to 14 days after stimulation. Importantly, under such culture conditions, we observed the highest fold change in the expression of mRNA corresponding to Runx2 as well as Col1A, at every analysed experimental time point (Figure 8a). Hypoxic microenvironment (2% O_2_) also stimulated the expression of a gene encoding osteopontin on day 7 in DPSCs in 2D culture (Figure S2). Recent evidence indicates that osteopontin regulates matrix remodelling and tissue calcification and may also be implicated in the pathophysiological process such as osteoporosis [47]. After seven days of differentiation, despite the increased expression of Runx2, we did not observe prominent calcium phosphate deposits in cells cultured in an environment containing 2% O_2_. The most explicit deposits of calcium phosphate were observed after 21 days of differentiation: in case of 3D culture, we observed large rounded deposits of calcium phosphate especially in the presence of 2% O_2_, whereas in case of 2D culture, these deposits were more prominent in the presence of approximately 18% O_2_ (Figure 8b).

Taken together, the results indicate that 3D cell encapsulation as well as the low concentration of O_2_ resembling conditions in the stem cell niches may favour osteogenic differentiation of DPSCs in an in vitro environment.

## 3. Discussion

Recent evidence indicates that dental pulp harbours a population of stem cells, such as DPSCs [43]. Nowadays, specific markers that may allow to uniquely define the immunophenotype of DPSCs are still unknown [48]. However, these cells express antigens that are characteristic of “classic” MSCs derived from mesodermal tissues. Such cells do not express hematopoietic markers [49]. Importantly, DPSCs were also shown to exhibit the expression of stemness-associated markers, such as *Oct-4, Nanog* and *Sox-2* [50] along with vimentin, as well as neural-specific markers such as nestin, N-tubulin and neurogenin-2 [51]. Due to the previously described unique features of such cells, one of the aims of this study was to analyse differentiation potential and other essential functional and morphological features of DPSCs in comparison with UC-MSCs. UC-MSCs may be used for allogenic or autologous applications. Moreover, accumulating evidence indicates that the transcriptomic profile of UC-MSCs indicating the expression of stemness- and bone development-related genes is similar to BM-MSCs. There is a possibility of elimination of restrictions that are characteristic for autologous BM-MSC. This might result in the replacement of these cells that are considered as gold standard for MSC research and clinical applications [16,17,52]. In the second phase of the current study, we focused on the osteogenic differentiation of DPSCs in 3D cultures in the hypoxic microenvironment resembling the stem cell niche.

We isolated DPSCs from dental pulp extracted from permanent healthy teeth of donors, whereas UC-MSCs were isolated from Wharton’s Jelly of UC. Isolated DPSCs represent an adherent fraction of dental pulp tissue and are spindle-shaped, fibroblast-like cells similar in morphology to UC-MSCs. By employing flow cytometry platform, we established the antigenic profiles of DPSCs containing the expression of CD45^−^/CD14^−^/CD34^−^/CD29^+^/CD44^+^/CD73^+^/CD90^+^/CD105^+^/Stro-1^+^/HLA-DR^−^. The immunophenotype of DPSCs is similar to the antigenic profile of UC-MSCs. Subsequently, by employing quantitative RT-PCR and histochemical staining, we confirmed that DPSCs, as well as UC-MSCs exhibit the capacity for tri-lineage mesenchymal differentiation. Collectively, DPSCs, as well as UC-MSCs, fulfil the classification criteria of MSCs published by ISCT [3]. It is important to emphasize that some investigators have pointed out that the exact status of DPSCs as MSCs is still not fully defined and requires further investigation [48]. Based on the fact that the typical characteristics and immunophenotype of DPSCs fulfil MSC classification criteria, DPSCs may be considered as a population of MSC with certain unique properties resulting from their developmental origin.

Due to the ectomesenchymal origin [53], the DPSCs may be effectively differentiated into functionally active neurons [51,54] demonstrating an attractive prospect for the treatment of injuries of the nervous system. However, the comparisons between the differentiation potential of DPSCs and other “classic” MSCs toward mature cell types other than neural cells have never been performed. Thus, in the current study, we compared the differentiation capacity of DPSCs with UC-MSCs isolated from human neonatal tissue, focusing on osteogenic, chondrogenic, adipogenic, cardiomyogenic, and angiogenic differentiation in vitro. In the case of osteogenic differentiation, the expression levels of mRNA for *Runx2* and osteocalcin were comparable between the DPSCs and UC-MSCs, whereas the expression of osteopontin was elevated in the UC-MSCs notably on the latter days following stimulation (14d). Other studies have shown that osteopontin may be expressed in many organs, with or without matrix, suggesting that this molecule may act as a structural molecule or humoral factor or cytokine and may not be a specific marker of osteogenesis [45], which may explain the elevated expression observed in UC-MSCs. Despite the comparable expression level of osteogenesis associated genes between DPSCs and UC-MSCs, histochemical staining demonstrated larger deposits of calcium phosphate following DPSC osteogenic stimulation in comparison with those in the stimulated UC-MSCs, which may indicate higher osteogenic capacity of DPSCs. Deposition of calcium phosphate, a bone mineral, is a characteristic for osteoblasts [55]. Since some investigators indicate a poor correlation between mRNA and protein expression levels, it may be necessary to extend the studies examining mRNA and protein expression in DPSCs in comparison with UC-MSCs to explain the observed higher deposition of calcium phosphate in DPSCs following osteogenic stimulation despite the low expression of mRNA [56]. Osteogenic differentiation of DPSCs was also confirmed by other investigators [57]. A higher alkaline phosphatase activity has been detected in human DPSCs cultured in osteogenic differentiation medium than that in BM-MSCs [11].

Interestingly, we also confirmed the chondrogenic potential of DPSCs and UC-MSCs. We observed higher expression of *Sox9, Acan*, and *Col10A1* mRNA in UC-MSCs, whereas the expression of *Col2A1* was comparable between both groups of SCs. Histochemical staining of DPSCs and UC-MSCs indicated the extracellular secretion of sulphated proteoglycans by both types of cells. Kinetics of differentiation was also similar between the analysed populations of SCs. Recent evidence indicates that *Col10A1* is a marker of hypertrophic chondrocytes, which may be implicated in bone growth. In contrast, it has also been observed in skeletal dysplasia and osteoarthritis disorders [44]. Thus, further investigation may be necessary to confirm the proper chondrogenic differentiation of UC-MSCs as well as DPSCs. Some investigators, however, have confirmed the higher chondrogenic potential of BM-MSCs in comparison to DPSCs [11]. Moreover, we observed a higher level of adipogenesis in UC-MSCs than that in DPSCs following stimulation, as measured by adipogenic gene expression at mRNA level. Both MSC populations secrete oil droplets, a characteristic of the ongoing adipogenic differentiation [58]. A lower adipogenic potential of DPSCs in comparison with BM-MSCs has also been demonstrated by Guo et al. [11].

In the next step, we analysed differentiation potential of DPSCs toward cells with cardiac and endothelial phenotype. After cardiomyogenic stimulation, we observed higher expression of *Gata-4, Nkx2.5*, and *Myl2c* mRNA in UC-MSCs than in DPSCs, which was confirmed by immunocytochemical staining. In the case of endothelial cell formation, elevated expression of angiogenesis-related genes (*Gata-2, Tie2*, VE-cadherin) was observed in UC-MSCs after seven days of stimulation with VEGF, whereas DPSCs were typically unresponsive toward such proangiogenic stimulation. The results suggested q limited switch towards cardiac and endothelial phenotype in UC-MSCs, while the signs of differentiation were hardly detected in DPSCs. However, other investigators have shown that in the co-culture of DPSCs with neonatal rat cardiomyocytes, the expression of cardiac-specific markers (including troponin I and β-myosin heavy chain) was increased in DPSCs shortly after co-incubation, suggesting a possible transient phenotypical switch into cardiac lineage under certain circumstances [59].

Since DPSCs may be effectively differentiated into osteoblasts, we also encapsulated these cells in the hydrogel matrix to create a 3D culture niche mimicking tissue and organ-specific microarchitecture to conduct osteogenic differentiation in vitro. Nevertheless, we observed a greater relative proliferation of DPSCs in 2D culture in comparison with 3D culture. Moreover, contrary to our initial expectations, we did not observe any influence of oxygen concentration on the proliferation ratio of DPSCs in both 2D and 3D culture at predetermined intervals. However, the increased proliferation of DPSCs in 2D culture was correlated with higher metabolic activity, whereas DPSCs in 3D culture exhibited lower metabolic activity correlating with lower proliferation and higher differentiation capacity in such conditions. It has been demonstrated that 3D culture allows the cells to maintain a better cell-to-cell contact and intercellular signalling network [60]. In the case of a hypoxic environment, we set up oxygen concentration equal to 2%, which was in the range of O_2_ concentration measured by other investigators in murine BM (1.54–2.0%) [61]. For a normoxic culture environment, Wegner et al. showed that the O_2_ concentration should be approximately 18% [62]. Hence, we assumed that the O_2_ concentration in the normoxic environment was approximately 18%. The typical mammalian cell culture conditions inside an incubator also include the temperature of 37 °C, 95% relative humidity and CO_2_ concentration of approximately 5%.

Kwon et al. has shown increased proliferation rate and percentage of DPSCs in S-phase under 5% hypoxic culture environment from day 5 of the culture accompanied with enhanced osteogenic differentiation capacity of these cells in comparison with the normoxic environment [63], whereas Iida et al. observed the inhibition of the osteo/odontogenic differentiation capacity of such cells, despite the enhanced proliferation rate of DPSCs under hypoxic environment [64]. Results related to the proliferation of DPSCs cultured under hypoxia have been controversial, which may be attributed to the lack of standardization of cell culture protocols [65]. In this study, we cultured DPSCs in the presence of 2% O_2_, whereas other investigators cultured cells in the presence of 5% or 3% O_2_ [63,64].

A decrease in cell proliferation might often be accompanied by the induction of cell differentiation [66] and 3D culture may facilitate the developmental processes allowing the cells to differentiate into more complex structures [67]. Thus, we analysed the osteogenic differentiation of DPSCs in 3D cultures under hypoxic or normoxic conditions.

We also analysed the osteogenic differentiation of DPSCs in 3D culture under hypoxic and normoxic conditions. The expression of mRNA corresponding to Runx2–osteogenesis regulating transcription factor-was elevated by approximately thrice the initial amount in DPSCs in 2D and 3D culture in the presence of 2% O_2_, compared to that in the undifferentiated cells. The elevated expression of *Runx2* in a 3D culture in an environment containing 2% O_2_ persisted up to day 14, suggesting long-term osteogenic induction. We observed the highest increase in the expression of early and late osteogenic markers such as *Runx2* and *Col1A*, respectively, at every interval in 3D culture in microenvironment under hypoxia than that in the 3D culture in a normoxic microenvironment. In the case of 3D culture, we also observed large rounded deposits of calcium phosphate, primarily in the presence of hypoxia.

Taken together, our results indicate that 3D cell encapsulation as well as low concentrations of oxygen may favour the osteogenic differentiation of DPSCs in vitro. The culture of human MSCs under hypoxic conditions enhances the differentiation into osteocytes. These cells produce more osteomatrix and the expression of osteogenesis-related genes (e.g., *Runx2, ALP*) is up-regulated [68,69]. Burakova et al. suggested that the effect of hypoxia on MSCs may be driven by specific factors explaining the opposite influence of low oxygen concentration observed by many investigators [70]. Recent evidence indicates that the 3D scaffold may have a major impact on the proliferation and activation of MSCs, whereas the 3D architecture supports the osteogenic differentiation of MSCs and enhances the production of mineralized bone matrix [71]. Increased expression of *Runx2* and *Col1A1* characterizing osteogenic differentiation of DPSCs in 3D culture under hypoxic environment observed in our study is consistent with recent evidence demonstrated by other investigators, confirming that the differentiation of MSCs encapsulated in alginate beads significantly increases the osteogenic differentiation of these cells in vitro [35]. Moreover, in the novel bone development or bone regeneration, vasculature delivers nutrients, growth factors, minerals, and oxygen. The scaffolds for bone regeneration should allow endothelial cell migration and the growth of new vessels, which may induce VEGF expression promoting the migration and proliferation of endothelial cells and positively influence the bone regeneration process [72]. After the co-culture of DPSCs and endothelial cell line EA.hy926 on chitlac-coated thermosets, an increased proliferation with enhanced DPSC osteogenic differentiation and EA.hy926 vessel formation was observed [73], confirming positive feedback of both cell fractions.

In our experiments, we used primary human DPSCs isolated from dental pulp derived from the teeth of three donors, whereas UC-MSCs were isolated from two umbilical cords. Moreover, for qRT-PCR analysis, every sample (prepared for each DPSCs line from each donor) was run in duplicate, whereas for the proliferation and metabolic activity assessment, every sample was run in triplicate. In some cases, we observed a high error of the mean (SEM), which may be the result of biological variability related to primary human tissues obtained from various donors. Data published by other investigators indicate that the DPSCs isolated from the patients at different ages exhibit different doubling time, expression of CD29 antigen, and percentage of apoptotic cells during propagation along with differentiation potential [74], which may have an additional impact on the variability of the cell response. Moreover, the DPSCs isolated from different teeth may possess varied differentiation capacity or immunomodulatory potential [75]. The high variation of the response and data presented in our study is not unusual during the analyses of SCs, including DPSCs. Other investigators have also demonstrated highly variable data from analysis of different populations of SCs, including DPSCs (results with high SEM values) [76]. Therefore, further investigations are required to find a correlation between the type of dental pulp derived from different teeth, the age of the donor, and the biological potential of DPSCs. Proper protocols might be developed for the preparation of optimal DPSC fraction as a product for advanced therapy with high pro-regenerative potential.

To summarize, we observed that human DPSCs possess a higher potential for osteogenic differentiation and similar chondrogenic and adipogenic differentiation potential compared to the “classic” MSCs such as UC-MSCs but a limited ability for the cardiac or endothelial phenotype in vitro. Moreover, the hypoxic environment especially in 3D culture conditions may enhance the osteogenic capacity of DPSCs in vitro. DPSCs encapsulated in injectable hydrogel might be a candidate for innovative advanced therapy for the treatment of bone (including alveolar bone) loss.

## 4. Materials and Methods

### 4.1. Isolation of Human DPSCs

The DPSCs were isolated from the dental pulp of human permanent healthy teeth, which were extracted based on the orthodontic indications in the Department of Oral Surgery, Faculty of Medicine, Jagiellonian University Medical College in Krakow and then donated for scientific research following approval by the Bioethics Committee at the Jagiellonian University in Krakow (approval number: 1072.6120.41.2017).

In the first step of isolation of DPSCs, the pulp chamber was exposed using the pulp drill (size: D Ø: 0.25 mm and 0.30 mm) to extract the dental pulp. Subsequently, the pulp chamber was gently rinsed with phosphate-buffered saline (PBS; GE Healthcare Life Sciences HyClone Laboratories, Malborough, MA, USA) containing 100 IU/mL penicillin and 100 µg/mL streptomycin solutions (Gibco, ThermoFisher Scientific, Waltham, MA, USA) to wash out the remaining pulp tissue. Following the mechanical disruption, the isolated pulp tissue was subjected to further enzymatic digestion using a mixture of collagenase I (3 mg/mL, Sigma-Aldrich, St. Louis, MO, USA) and dispase (4 mg/mL, Sigma-Aldrich) for 30 min at 37 °C. The enzymes were inactivated by adding a complete cell culture medium (DMEM/F12 supplemented with 10% FBS, Sigma-Aldrich; and 100 IU/mL penicillin, 100 µg/mL streptomycin, Gibco, ThermoFisher Scientific). Released cells as well as the larger pieces of dental pulp were washed and seeded on 24-well culture plates favouring primary cell adhesion (Corning Primaria Culture Plate; Falcon Coring, Tewksbury, MA, USA) and were further cultured under standard conditions (37 °C, 5% CO_2_, 95% humidity). Fresh culture medium was added after 24 h post-seeding. The tissue pieces were removed after 10 days and the adherent cells were washed with PBS and cultured in the complete cell culture medium. The scheme of isolation of DPSCs is presented in Figure 1a. DPSCs were passaged with 0.25% trypsin/EDTA (Gibco, ThermoFisher Scientific) when the confluence of cells reached close to 80–90%. The cells were isolated from three separate teeth derived from three individual donors and were further cultivated individually as separate isolates of DPSCs. DPSCs collected from a single human donor were used in every experiment. Thus, three independent DPSC lines derived from three teeth that were harvested from three individual human donors were used to replicate the experiments.

### 4.2. Isolation of Human UC-MSCs

The UC-MSCs were isolated from human umbilical cords obtained from The Polish Stem Cell Bank, with permissions from the Polish Ministry of Health (MZ-PZ-TSZ-025-15906-36/AB/14). The umbilical cord was washed with PBS to remove the remaining blood. Subsequently, the vein and arteries were dissected, the Wharton’s jelly tissue was cut into 1–2 mm pieces and placed on tissue culture dishes (Falcon) containing complete cell culture medium (DMEM/F12 supplemented with 10% FBS, Sigma-Aldrich,; and 100 IU/mL penicillin, 100 µg/mL streptomycin, Gibco, ThermoFisher Scientific). The tissue pieces were removed after 5 days, the adherent cells were washed with PBS and cultured in the complete cell culture medium. The scheme of isolation of UC-MSCs is presented in Figure 1b. The cells were cultured under standard cell culture conditions. UC-MSCs were passaged with 0.25% trypsin/EDTA (Gibco, ThermoFisher Scientific) when the confluence of cells reached close to 80–90%. The cells isolated from the Wharton’s Jelly tissue from the UC of every donor were cultivated separately. In the three replicated experiments, two independent UC-MSC lines derived from the two Wharton’s jelly tissues obtained from UCs collected from two separate human donors were used.

### 4.3. Cell Counting and Viability Assessment

To assess the number of cells and examine their viability, the cell suspension was mixed with 0.2% Trypan Blue Stain (ThermoFisher Scientific), placed in cell counting slides, and analysed by Countess Automated Cell Counter (ThermoFisher Scientific).

### 4.4. Antigenic Phenotyping by Flow Cytometry

To compare the phenotype of DPSCs and UC-MSCs, the cells of both fractions were resuspended in standard staining medium (DMEM/F12 supplemented with 2% FBS; both from Sigma-Aldrich, and were further immunolabelled with the following monoclonal antibodies against human antigens: anti-CD45 (FITC, clone: HI30, Biolegend, San Diego, CA, USA), anti-CD14 (FITC, clone: MφP9, BD Biosciences, Franklin Lakes, NJ, USA), anti-CD34 (FITC, clone: 581, BD Biosciences, Franklin Lakes NJ, USA), anti-CD29 (PE/Cy5, clone: TS2/16, Biolegend), anti-CD44 (PE, clone: BJ18, Biolegend), anti-CD73 (PE, clone: AD2, Biolegend), anti-CD90 (PE, clone: 5E10, Biolegend), anti-CD105 (PE, clone: 43A3, Biolegend), anti-Stro-1 (Alx647, clone: STRO-1, Biolegend,) and anti-HLA-DR (PE, clone: L243, Biolegend). For each analysed antigen, appropriate isotype control was used as following: mouse IgG1 (FITC, clone: MOPC-21, BD Biosciences, mouse IgG2 (Alx488, clone: 133303, R&D Systems, Minneapolis, MN, USA), mouse IgG1 (PE/Cy5, clone: MOPC-21, BD Biosciences,), mouse IgG1 (PE, clone: MOPC-21, BD Biosciences,), mouse IgG2 (PE, clone: IS6-11E5.11, Miltenyi Biotec, Bergisch Gladbach, Germany) and mouse IgG1 (APC, clone: IS5-21F5, Miltenyi BiotecStaining was performed for 30 min at 4 °C according to the manufacturer’s protocols. Cells were further washed and analysed using LSR Fortessa flow cytometer and FACS Diva software (Becton Dickinson, Franklin Lakes, NJ, USA).

### 4.5. Differentiation of DPSCs and UC-MSCs

#### 4.5.1. Osteogenic, Chondrogenic, and Adipogenic Differentiation

Plates with 12-wells were coated with 0.1% gelatin (Sigma-Aldrich). In the case of osteogenic and adipogenic differentiation, 2.0 × 10^4^ cells were seeded per well in the complete cell culture medium (DMEM/F12 supplemented with 10% FBS, Sigma-Aldrich; and 100 IU/mL penicillin, 100 µg/mL streptomycin, Gibco, ThermoFisher Scientificto reach 60% confluence. Subsequently, the medium was replaced with complete StemPro Osteogenesis Differentiation Kit or StemPro Adipogenesis Differentiation Kit (Gibco, ThermoFisher Scientific), stimulating osteogenic or adipogenic differentiation, respectively. The cultures were refed every 3–4 days. In the case of chondrogenic differentiation, micro mass cultures were generated by seeding 5 µL droplets of cell solution (1.6 × 10^7^ viable cells/mL) and were incubated for 2 h under high humidity conditions. Next, the micro masses were flooded with the complete cell culture medium. After 24 h, the medium was changed to StemPro chondrogenesis differentiation medium (Gibco, ThermoFisher Scientific). The cultures were re-fed every 2–3 days.

Cells were examined for osteogenic, chondrogenic, and adipogenic differentiation on days 7, 14 and 21 of culture, following histochemical staining performed for identifying the cell phenotype.

#### 4.5.2. Cardiomyogenic Differentiation

12-well plates were coated with 50 μg/mL collagen type I (Sigma-Aldrich), and 2.0 × 10^4^ cells were seeded per well in complete cell culture medium (DMEM/F12 supplemented with 10% FBS, Sigma-Aldrich; and 100 IU/mL penicillin, 100 µg/mL streptomycin, Gibco, ThermoFisher Scientific) for 24 h. The following day, the culture medium was changed with a cardiomyogenesis-stimulating medium containing DMEM/F12 supplemented with 2% FBS (both from Sigma-Aldrich) and 10 ng/mL bFGF, 10 ng/mL VEGF and 10 ng/mL TGF-β1 (all growth factors were from Peprotech, London, UK). The growth factors were supplemented daily and the whole medium was replaced every two days. Cells were examined for cardiac differentiation on days 7, 14 and 21 of culture, following the staining for cardiac-specific markers.

#### 4.5.3. Endothelial Differentiation

Twelve-well plates were coated with a solution containing 50 μg/mL fibronectin (BD Bioscience) and 0.1% gelatin (Sigma-Aldrich). Cells were seeded with the density of 2.0 × 10^4^ cells per well in the complete cell culture medium (DMEM/F12 supplemented with 10% FBS, Sigma-Aldrich; and 100 IU/mL penicillin, 100 µg/mL streptomycin, Gibco, ThermoFisher Scientific) for 24 h. The following day, the culture medium was changed with EGM-2MV Endothelial Cell Growth Medium (Lonza, Basel, Switzerland). EGM-2MV was replaced every two days. Cells were examined for endothelial differentiation on days 7, 14 and 21 days of culture, following staining for endothelial markers.

The expression of selected osteogenic, chondrogenic, adipogenic, cardiomyogenic and angiogenic-specific markers was evaluated by measuring the levels of corresponding mRNAs as well as by histochemical or immunocytochemical staining.

### 4.6. Gene Expression Analysis by Real-Time RT-PCR

Total RNA was isolated using the GeneMATRIX Universal RNA Purification Kit (Eurx, Gdansk, Poland) according to the manufacturer’s protocol for RNA isolation from cell cultures. Briefly, to lyse the cells, lysis buffer (Eurx) with 1% Bond-Breaker reagent (ThermoFisher Scientificwas used. RNAs were treated with Turbo DNase (Ambion, ThermoFisher Scientificto remove DNA contamination. RNA concentration was determined by Nano Photometer^®^ device (Implen, Munich, Germany). Purified RNAs were stored at −80 °C.

cDNA synthesis was performed using NG dART RT kit (EURx, Gdansk, Poland) according to the manufacturer’s protocol with the following conditions: one cycle at 25 °C for 10 min, one cycle at 50 °C for 40 min, and one cycle at 85 °C for 5 min by employing C1000 Touch™ Thermal Cycler (Bio-Rad, Hercules, CA, USA). The samples of cDNAs were stored at −20 °C for further analysis. Expression of selected human genes associated with osteogenic (Osteocalcin, Osteopontin, *Runx2*), chondrogenic (*Acan, Col10A1, Col1A1, Sox9*), adipogenic (*CEBPα, PPARγ*), cardiomyogenic (*Gata-4, Myl2c, Nkx2.5*) or endothelial (*Gata-2, Tie-2*, VE-cadherin) differentiation were examined by real-time PCR using an ABI PRISM 7000 sequence detection system (Applied Biosystems, ThermoFisher Scientific, Waltham MA, USA). β2-microglobulin was used as a control housekeeping gene.

Real-time PCR was performed using SYBR Green qPCR Master Mix (EURx), cDNA template (10 ng), forward primer (1 µM) and reverse primer (1 µM; both from Genomed, Warsaw, Poland). The sequences of primers used are included in Table 1. Reactions were performed under the following conditions: one cycle at 50 °C for 2 min, one cycle at 95 °C for 10 min, followed by 40 cycles at 94 °C for 15 s, 60 °C for 30 s and 72 °C for 30 s. Relative quantification of genes expression was calculated using the comparative ddC_t_ method.

### 4.7. Histochemical Staining

On days 7, 14 and 21 of osteogenic, chondrogenic and adipogenic differentiation, the cells were washed with PBS and fixed with 4% paraformaldehyde (POCH, Avantor Performance Materials Poland S.A., Gliwice, Poland) for 30 min at RT.

To evaluate calcium phosphate deposition in the cells differentiated into osteoblasts, the cells were rinsed twice with distilled water following fixation and stained with 2% Alizarin Red S solution with pH 4.2 (Sigma-Aldrich) for 3 min.

To visualize sulphated proteoglycans that are characteristic for chondrogenic differentiation, fixed cells were rinsed with PBS and stained with 1% Alcian Blue solution (Sigma-Aldrichprepared in 0.1 N HCl (POCH, Avantor Performance Materials Poland S.A) for 30 min. The wells were rinsed thrice with 0.1 N HCl (POCH) and then distilled water was added to neutralize the acidity.

To visualize the presence of oil droplets during adipogenic differentiation, the cells were rinsed with distilled water and incubated with 60% isopropanol (POCH, Avantor Performance Materials Poland S.A) for 5 min. After the removal of isopropanol, the cells were stained with 1% Oil Red O solution (Sigma-Aldrich) for 5 min.

After histochemical staining, the cells were rinsed with distilled water and visualized using an Olympus IX81 microscope equipped with MicroPublisher 3.3 RTV camera (Olympus, Tokyo, Japan).

### 4.8. Immunocytochemistry

Immunocytochemistry staining was performed to evaluate (i) Gata-4 and troponin T-C expression in DPSCs and UC-MSCs differentiated into cardiomyocytes as well as (ii) Gata-2 and VE-cadherin expression in angiogenic differentiation. For this purpose, on days 7, 14 and 21 of the differentiation culture, the medium was removed, cells were washed with PBS (GE Healthcare Life Sciences HyClone Laboratories, Malborough, MA, USA), and fixed with 4% paraformaldehyde (POCH, Avantor Performance Materials Poland S.A.) for 20 min at RT. Cells were subsequently washed thrice with PBS, permeabilized with 0.1% Triton X-100 solution (Sigma-Aldrich) for 8 min at RT, and washed again thrice with PBS. Subsequently, cells were stained against: (i) cardiac-specific proteins–with primary anti-Gata-4 antibody (mouse monoclonal IgG_2a_, 1:50; Santa Cruz Biotechnology, Dallas, TX, USA) and anti-troponin T-C antibody (goat polyclonal IgG, 1:20; Santa Cruz Biotechnology) (ii) endothelial-specific proteins–with primary anti-Gata-2 (rabbit polyclonal IgG, 1:50; Santa Cruz Biotechnology) and anti-VE-cadherin (mouse monoclonal IgG_1_, 1:20; Santa Cruz Biotechnology) for 16 h at 4 °C. For (i) cardiac and (ii) endothelial markers detection, the following secondary antibodies were subsequently added, respectively: (i) donkey anti-mouse IgG antibody conjugated with Alexa Fluor 488 (1:250; Jackson ImmunoResearch, Cambridgeshire, UK) and donkey anti-goat antibody conjugated with Alexa Fluor 546 (1:250; ThermoFisher Scientific) (ii) goat anti-mouse IgG antibody conjugated with Alexa Fluor 546 (1:250; ThermoFisher Scientific) and goat anti-rabbit IgG antibody conjugated with Alexa Fluor 488 (1:250; ThermoFisher Scientific). The concentrations of the used primary and secondary antibodies were selected based on the optimization experiments conducted previously. Moreover, appropriate IgG controls were used to confirm the specificity of antibodies prior to the current study. Staining with secondary antibodies was performed for 2 h in 37 °C protected from light. Cells were further washed with PBS and nuclei were stained with DAPI (2 µM, ThermoFisher Scientific) for 15 min in 37 °C protected from light. VECTASHIELD Mounting Medium (Vector Laboratories, Burlingame, CA, USA) was used to mount coverslips. The preparations were analysed with Leica DMI6000B ver. AF7000 fluorescent microscope (Leica Microsystems GmbH, Welzlar, Germany). The control (undifferentiated) DPSCs and UC-MSCs were also stained with primary and secondary antibodies according to the protocol described above, and the results are presented in Figure S1.

### 4.9. Mechanical Characterization of the Hydrogel Matrix

Mechanical properties of BD PuraMatrix Peptide Hydrogel (Corning, Tewskbury, MA, USA) were investigated using atomic force microscopy (AFM, CellHesion head, JPK Instruments, Berlin, Germany) in force mapping mode. To probe hydrogel samples, commercially-available silicon nitride cantilevers (MLCT-C, Bruker, Billerica, MA, USA) with a nominal spring constant of 0.01 N/m were applied. Force curves, i.e., dependencies between cantilever deflection and relative sample position, were acquired over a grid of 8 × 8 pixels within a scan area of 50 × 50 μm. The maximum load force (F) was 5 nN and load speed of 8 μm/s was maintained. Young’s modulus was determined using Hertz contact mechanics as described previously [77]. Briefly, the following relation between the load force (F) and resulting indentation (Δz) for the paraboloidal assumption of the probing tip was applied:

(1) F=4·R·E3·(1−v2)·Δz32

**Theorem** **1.** *Maximum load force (F). In this theorem, R is the radius of tip curvature, E is Young’s modulus and v is the Poisson ratio of the material (here assumed to be 0.5 treating hydrogels as incompressible material).*

JPK Data Processing software was used to apply this equation to the experimental data to obtain the value of Young’s modulus for each force curves. The final Young’s modulus was obtained by averaging all force curves and was expressed as a mean and standard deviation.

### 4.10. 3D Encapsulation of DPSCs within the Hydrogel Matrix

BD PuraMatrix Peptide Hydrogel (Corning) with a concentration of 1% (*w*/*v*) after decreasing the viscosity by vortexing was diluted with a cell culture-grade water (PAA,) to the concentration of 0.3%. The cells re-suspended in a 20% sucrose solution (Sigma-Aldrich) were centrifuged at 320× *g* for 7 min at RT to wash out residual salts from the culture medium (to prevent early hydrogel gelation). After centrifugation, the cells were re-suspended in a 20% sucrose solution, and the cell number and viability were assessed by Countess Automated Cell Counter as described in Section 4.3. To obtain a final hydrogel concentration of 0.15%, 0.3% hydrogel was diluted with the same volume of cell suspension in 20% sucrose solution (at 2× the final desired cell concentration). Thus, the final concentrations of hydrogel and sucrose were equal to 0.15% and 10%, respectively. The whole volume was carefully mixed and added to the centre of each well without introducing bubbles. The following volumes of the mixtures were used: 50 μL/well in a 96-well plate, 250 μL/well in a 24-well plate, and 400 μL/well in a 12-well plate (Corning) and are presented in Table 2. The gelation of the BD PuraMatrix hydrogel was initiated by the addition of the complete cell culture medium (DMEM/F12 supplemented with 10% FBS, Sigma-Aldrich; and 100 IU/mL penicillin, 100 µg/mL streptomycin, Gibco, ThermoFisher Scientific) and by gently running culture media down the side of the well on top of the hydrogel. Within 1 h post 3D cell encapsulation in hydrogel and following their gelation, 70% of the medium was changed twice to stabilize pH. The DPSCs were cultured for two days under standard culture conditions.

### 4.11. 3D and 2D Culture of DPSCs in Vitro

To establish a 2D culture of the DPSCs, cell culture plates (Corning) were coated with 0.1% gelatin (Sigma-Aldrich). The cell suspension was prepared in 20% sucrose solution and seeded at a concentration of 2 × 10^4^/well in a 12-well plate in the complete cell culture medium (DMEM/F12 supplemented with 10% FBS, Sigma-Aldrich; and 100 IU/mL penicillin, 100 µg/mL streptomycin, Gibco, ThermoFisher Scientific, Waltham, MA, USA). DPSCs were cultured for two days under standard culture conditions. DPSCs encapsulated in the 3D hydrogel or seeded on 2D gelatin-coated cell culture plates were further cultured at 37 °C in a humidified atmosphere containing 5% CO_2_ and the following oxygen concentrations were used: (i) 2% of O_2_ (hypoxia) or (ii) about 18% of O_2_ (normoxia) for 7, 14 and 21 days.

### 4.12. Assessment of DPSC Proliferation and Metabolic Activity In Vitro

The proliferation and metabolic activity of DPSCs in 3D or 2D culture in the environment containing 2% (hypoxia) or about 18% of O_2_ (normoxia) were measured by MTS assay or by analysing ATP content, respectively. The tests were performed every 24 h until seven days post cell encapsulation/seeding.

For MTS assay, DPSCs were encapsulated in the hydrogel or seeded onto gelatin-coated surfaces of 96-well transparent plates (Corning, Tewskbury MA, USA) at a density of 10^3^ cells/well for 2D culture conditions and 2 × 10^3^ cells/well for 3D culture conditions. The analysis was performed using the Cell Counting Kit-8 (Sigma-Aldrich, St. Louis MO, USA). For this purpose, 50 µL of WST-8 reagent was added into each well and incubated for 4 h. The absorbance was measured at 450 nm wavelength using the Multiskan FC Microplate Photometer (ThermoFisher Scientific, Waltham MA, USA). For the measurement of ATP concentrations, DPSCs were encapsulated in the hydrogel or seeded onto gelatin-coated surfaces of 96-well white plates (Perkin Elmer, Waltham, MA, USA). Subsequently, the assay was conducted using the ATP Lite Luminescence assay kit according to the manufacturer’s instructions (Perkin ElmerLuminescence was measured using the Infinite^®^ M200PRO plate reader (Tecan, Mannedorf, Zurich, Switzerland).

### 4.13. Osteogenic Differentiation of DPSCs in 3D or 2D in Vitro Culture

Osteogenic differentiation of DPSCs in 3D or 2D cultures was initiated 1–2 days after the encapsulation of DPSCs in hydrogel (3D culture) or seeding of DPSCs into cell culture plates coated with 0.1% gelatin (2D culture). For this purpose, a StemPro Osteogenesis Differentiation Kit (ThermoFisher Scientific) was used. The differentiation medium was changed every 3–4 days. On days 7, 14 and 21 of osteogenic differentiation, DPSCs from 3D and 2D culture were recovered for the analysis of expression of osteogenesis-associated genes by Real-Time RT-PCR. Moreover, the presence of calcium phosphate deposits was confirmed by the staining of cells in the hydrogel or those seeded on the 2D surface by Alizarin Red S solution as described in Section 4.7. For 3D culture, a larger number of washes (approx. 4–6) were performed to reduce the red background staining of the hydrogel.

### 4.14. Recovery of DPSCs from the Hydrogel Matrix

The DPSCs were isolated from the hydrogel according to the Puramatrix Peptide Hydrogel manufacturer’s protocol titled “Cell recovery for sub-culturing or biochemical analyses” (Corning). Briefly, the hydrogel with the culture medium was mechanically disrupted by pipetting. The suspension was then transferred to a 15 mL centrifuge tube. The wells were washed with PBS (GE Healthcare Life Sciences HyClone Laboratories) to collect the remaining hydrogel fragments, transferred into a centrifuge tube, and centrifuged at 320× *g* for 7 min at RT. The hydrogel pellet was re-suspended in PBS (GE Healthcare Life Sciences HyClone Laboratories) and centrifuged at 320× *g* for 7 min at RT. Subsequently, the hydrogel pellet was re-suspended in 0.25% Trypsin/EDTA (Gibco; ThermoFisher Scientific) following digestion for approx. 10 min at 37 °C. The trypsin was inactivated with complete cell culture medium and the suspension was centrifuged at 320× *g* for 7 min at RT.

### 4.15. Statistical Analysis

Data are represented as mean ± SD or SEM as indicated. Statistical analyses were performed with Student’s *t*-test. *p* < 0.05 was considered statistically significant.

## 5. Conclusions

In conclusion, DPSCs exhibit: (i) higher osteogenic differentiation capacity, (ii) comparable chondrogenic and adipogenic differentiation potential and (iii) limited ability for the cardiac or endothelial phenotype in comparison with the other “classic” MSCs (UC-MSCs). The current results may help determine the future direction of the application of these cells in regenerative therapies.

Importantly, 3D cell encapsulation as well as the low concentration of O2 resembling conditions in the stem cell niches may favour osteogenic differentiation of DPSCs in an in vitro environment. The positive impact of hypoxia on the osteogenic potential of DPSCs was visible notably in 3D culture conditions.

Thus, tissue engineering approaches combining DPSCs, 3D biomaterial scaffolds, and other stimulating chemical factors may represent new innovative paths in the development of tissue repair.

## Figures and Tables

**Table 1:** List of primers employed in real-time RT-PCR.

Gene	Sequences
β2-microglobulin	(F) 5′ AATGCGGCATCTTCAAACCT 3′
	(R) 5′ TGACTTTGTCACAGCCCAAGATA 3′
ACAN	(F) 5′ AGGCAGCGTGATCCTTACC 3′
	(R) 5′ GGCCTCTCCAGTCTCATTCTC 3′
Sox-9	(F) 5′ TGGGCAAGCTCTGGAGACTTC 3′
	(R) 5′ ATCCGGGTGGTCCTTCTTGTG 3′
Col10A1-F	(F) 5′ GCAACTAAGGGCCTCAATGG 3′
	(R) 5′ CTCAGGCATGACTGCTTGAC 3′
Col2A1	(F) 5′ CGTCCAGATGACCTTCCTACG 3′
	(R) 5′ TGAGCAGGGCCTTCTTGAG 3′
Osteocalcin	(F) 5′ CGCTGGTCTCTTCACTAC 3′
	(R) 5′ CTCACACTCCTCGCCCTATT 3′
Osteopontin	(F) 5′ ACTCGAACGACTCTGATGATGT 3′
	(R) 5′ GTCAGGTCTGCGAAACTTCTTA 3′
Runx2	(F) 5′ GGAGTGGACGAGGCAAGAGTTT 3′
	(R) 5′ AGCTTCTGTCTGTGCCTTCTGG 3′
PPARγ	(F) 5′ AGGCGAGGGCGATCTTGACAG 3′
	(R) 5′ GATGCGGATGGCCACCTCTTT 3′
CEBPα	(F) 5′ AGGTTTCCTGCCTCCTTCC 3′
	(R) 5′ CCCAAGTCCCTATGTTTCCA 3′
GATA-4	(F) 5′ AACGACGGCAACAACGATAAT 3′
	(R) 5′ GTTTTTTCCCCTTTGATTTTTGATC 3′
Nkx2.5	(F) 5′ TGCTGCTCACAGGGCCCGATACTTC 3′
	(R) 5′ TCCTTTCGAGCTCAGTGCACCACAAAAC 3′
hMyl2A-F	(F) 5′ GGGCCCCATCAACTTCACCGTCTTCC 3′
	(R) 5′ TGTAGTCGATGTTCCCCGCCAGGTCC 3′
Tie-2	(F) 5′ TCCCGAGGTCAAGAGGTGTA 3′
	(R) 5′ AGGGTGTGCCTCCTAAGCTA 3′
GATA-2	(F) 5′ GCTCGTTCCTGTTCAGAAGG 3′
	(R) 5′ GCCATAAGGTGGTGGTTGTC 3′
VE-cadherin	(F) 5′ TTTTCCAGCAGCCTTTCTACCA 3′
	(R) 5′ GCGGATGGAGTATCCAATGCTA 3′

**Table 2:** Preparing of 3D encapsulation of DPSCs within the hydrogel matrix.

3D Cell Encapsulation			
Type of Cell Culture Plate	Volume of Mixture: 0.15% Hydrogel + 10% Sucrose (Per Well)	Number of Encapsulated Cells	Volume of Added Cell Culture Media (for Gelation)
96-well	50 µL	2.0 × 103	100 µL
24-well	250 µL	5.0 × 104	500 µL
12-well	400 µL	1.0 × 105	800 µL

## Supplementary Materials

Supplementary Materials can be found at https://www.mdpi.com/1422-0067/21/17/6172/s1. Figure S1. Immunocytochemical staining of DPSCs and UC-MSCs at 7 day of standard cell culture. Figure S2. Expression of osteopontin during osteogenic differentiation of DPSCs encapsulated in hydrogel (3D) or seeded on the surface coated with gelatin (2D) cultured in hypoxic (2% O2) or normoxic (18% O2) environment. Table S1. Fold change in mRNA expression for osteogenesis related genes (osteocalcin, osteopontin, *Runx2*) in DPSCs and UC-MSCs by Real-Time RT-PCR. Table S2. Fold change in mRNA expression for chondrogenesis related genes (*Acan, Col10A1, Col2A1, Sox9*) in DPSCs and UC-MSCs by Real-Time RT-PCR. Table S3. Fold change in mRNA expression for adipogenesis related genes (*CEBPα, PPARγ*) in DPSCs and UC-MSCs by Real-Time RT-PCR. Table S4. Fold change in mRNA expression for cardiomyogenesis related genes (*Gata-4, Nkx2.5, Myl2c*) in DPSCs and UC-MSCs by Real-Time RT-PCR. Table S5. Fold change in mRNA expression for endothelial related genes (*Gata-2, Tie-2*, VE-cadherin) in DPSCs and UC-MSCs by Real-Time RT-PCR.

## Abbreviations

BM	Bone marrow
BM-MSCS	Bone marrow-derived mesenchymal stem/stromal cells
DPSCs	Dental pulp stem cells
ISCT	International Society for Cellular Therapy
MSCs	Mesenchymal stem/stromal cells
O_2_	Oxygen
SCs	Stem cells
UC-MSCs	Umbilical cord Wharton’s jelly-derived mesenchymal stem/stromal cells
2D	Two-dimensional
3D	Three-dimensional
