# Supplementary material for: Multilineage Differentiation Potential of Human Dental Pulp Stem Cells—Impact of 3D and Hypoxic Environment on Osteogenesis In Vitro
Source: Int J Mol Sci. 2020 Aug 26;21(17):6172. doi: 10.3390/ijms21176172 (PMC7504399; doi:10.3390/ijms21176172)
Supplement: Supplementary file 1 [file ijms-21-06172-s001.pdf]

**Supplementary materials**

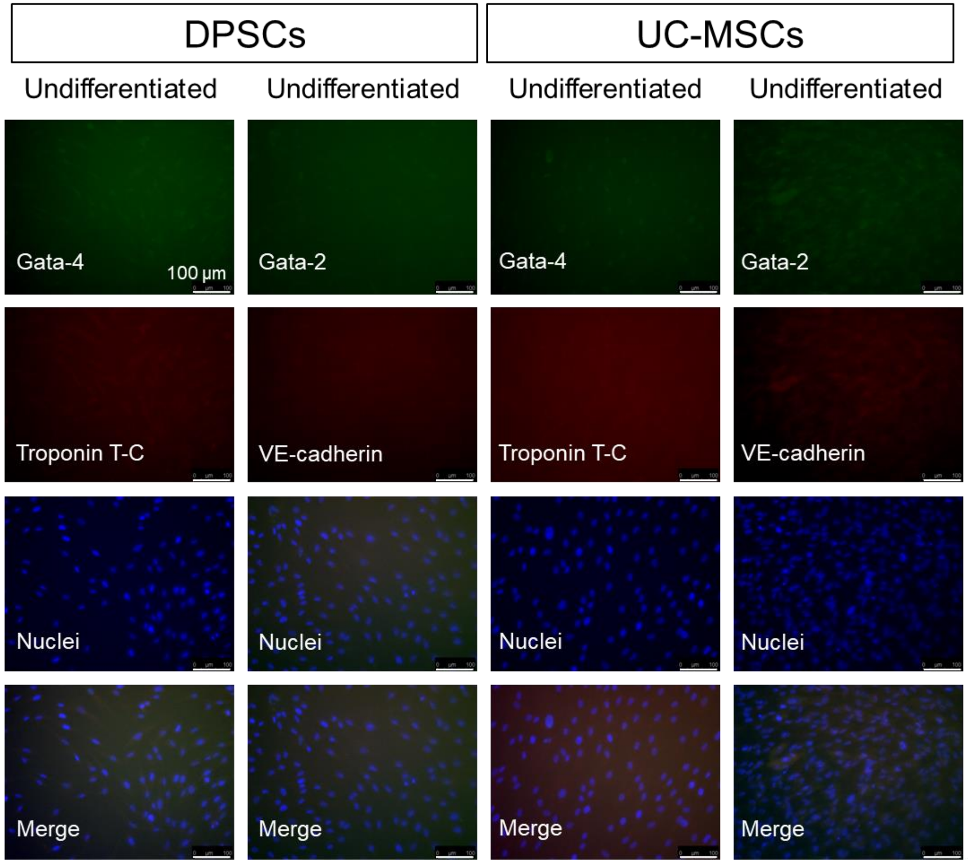


(a) (b)

**Figure S1.** Immunocytochemical staining of DPSCs and UC-MSCs at 7 day of standard cell culture. (a) Representative images of cardiomyogenic and endothelial marker expression in undifferentiated (control) DPSCs. (b) Representative images of cardiomyogenic and endothelial marker expression in undifferentiated (control) UC-MSCs. Cells were cultured in DMEM/F12 supplemented with 10% FBS. At 7 day. cells were fixed. permeabilized and stained against intranuclear transcription factor Gata-4 (Alexa Fluor 488. green) and troponin T-C (Alexa Fluor 546. red) or against intranuclear transcription factor Gata-2 (Alexa Fluor 488. green) and VE-cadherin (Alexa Fluor 546. red). Nuclei were co-stained with DAPI (blue). Cells were analyzed with Leica DMI6000B ver. AF7000 fluorescent microscope. Scale bars: 100 µm.

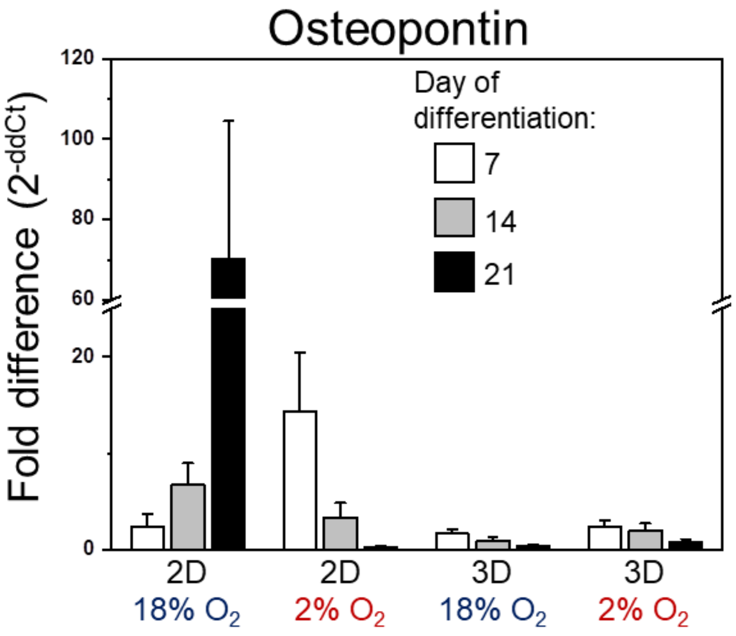


**Figure S2.** Expression of osteopontin during osteogenic differentiation of DPSCs encapsulated in hydrogel (3D) or seeded on the surface coated with gelatin (2D) cultured in hypoxic (2% $O_2$) or normoxic (18% $O_2$) environment. Quantitative analysis of mRNA expression for osteopontin in DPSCs at 7. 14 and 21 day of differentiation by Real-Time RT-PCR. Fold change in expression of analyzed gene in control cells before differentiation were calculated as 1.0. Results are presented as means±SEM. $n = 3$ (every sample w analyzed in duplicate). * $p < 0.05$ ($t$-test).

**Table S1.** Fold change in mRNA expression for osteogenesis related genes (osteocalcin. osteopontin. *Runx2*) in DPSCs and UC-MSCs by Real-Time RT-PCR. Grey color indicates value of change of fold change. The darker shades of gray correspond to a higher fold change in expression for tested gene.

| **DPSCs** | | Mean fold change | | | **Mean from 3 experiments** | SEM | | **UC-MSCs** | | Mean fold change | | | **Mean from 3 experiments** | SEM |
|---|---|---|---|---|---|---|---|---|---|---|---|---|---|---|
| Day | Gene | Sample 1 | Sample 2 | Sample 3 | | | | Day | Gene | Sample 1 | Sample 2 | Sample 3 | | |
| 7 | Osteocalcin | 0,23 | 0,02 | 0,98 | **0,41** | 0,29 | | 7 | Osteocalcin | 0,36 | 0,18 | 0,18 | **0,24** | 0,06 |
| 14 | | 0,27 | 0,76 | 0,49 | **0,51** | 0,14 | | 14 | | 0,98 | 0,65 | 0,49 | **0,71** | 0,15 |
| 21 | | 0,40 | 1,01 | 0,59 | **0,67** | 0,18 | | 21 | | 0,41 | 0,31 | 0,45 | **0,39** | 0,04 |
| 7 | Osteopontin | 0,64 | 0,03 | 2,18 | **0,95** | 0,64 | | 7 | Osteopontin | 12,99 | 4,66 | 2,17 | **6,61** | 3,27 |
| 14 | | 0,22 | 0,12 | 1,10 | **0,48** | 0,31 | | 14 | | 8,67 | 17,00 | 5,06 | **10,24** | 3,54 |
| 21 | | 0,56 | 0,05 | 3,45 | **1,36** | 1,06 | | 21 | | 4,44 | 5,75 | 1,10 | **3,76** | 1,39 |
| 7 | Runx2 | 1,63 | 1,02 | 0,98 | **1,21** | 0,21 | | 7 | Runx2 | 0,93 | 1,82 | 0,19 | **0,98** | 0,47 |
| 14 | | 2,41 | 1,02 | 1,20 | **1,55** | 0,44 | | 14 | | 2,17 | 1,54 | 0,91 | **1,54** | 0,36 |
| 21 | | 1,42 | 1,31 | 0,89 | **1,20** | 0,16 | | 21 | | 1,89 | 0,91 | 0,78 | **1,20** | 0,35 |

**Table S2.** Fold change in mRNA expression for chondrogenesis related genes (*Acan. Col10A1. Col2A1. Sox9*) in DPSCs and UC-MSCs by Real-Time RT-PCR. Grey color indicates value of change of fold change. The darker shades of gray correspond to a higher fold change in expression for tested gene.

| **DPSCs** | | Mean fold change | | | **Mean from 3 experiments** | SEM | | **UC-MSCs** | | Mean fold change | | | **Mean from 3 experiments** | SEM |
|---|---|---|---|---|---|---|---|---|---|---|---|---|---|---|
| Day | Gene | Sample 1 | Sample 2 | Sample 3 | | | | Day | Gene | Sample 1 | Sample 2 | Sample 3 | | |
| 7 | Acan | 0,07 | 0,06 | 2,45 | **0,86** | 0,80 | | 7 | Acan | 1,82 | 0,01 | 2,53 | **1,45** | 0,75 |
| 14 | | 0,01 | 0,17 | 0,18 | **0,12** | 0,06 | | 14 | | 8,01 | 0,04 | 0,52 | **2,86** | 2,58 |
| 21 | | 0,01 | 0,07 | 0,08 | **0,05** | 0,02 | | 21 | | 251,21 | 0,02 | 45,43 | **98,89** | 77,28 |
| 7 | Sox9 | 6,20 | 5,24 | 1,63 | **4,36** | 1,39 | | 7 | Sox9 | 261,13 | 0,30 | 16,03 | **92,49** | 84,44 |
| 14 | | 6,07 | 0,66 | 2,45 | **3,06** | 1,59 | | 14 | | 296,07 | 0,81 | 9,03 | **101,97** | 97,08 |
| 21 | | 0,65 | 0,41 | 0,59 | **0,55** | 0,07 | | 21 | | 903,49 | 0,12 | 49,12 | **317,58** | 293,30 |
| 7 | Col10A1 | 3,73 | 3,09 | 7,96 | **4,92** | 1,53 | | 7 | Col10A1 | 908,26 | 0,65 | 876,62 | **595,18** | 297,40 |
| 14 | | 1,51 | 0,63 | 2,22 | **1,45** | 0,46 | | 14 | | 670,02 | 3,73 | 251,71 | **308,48** | 194,43 |
| 21 | | 1,90 | 3,22 | 0,94 | **2,02** | 0,66 | | 21 | | 4011,96 | 1,13 | 3360,55 | **2457,88** | 1242,68 |
| 7 | Col2A1 | 8,00 | 5,27 | 0,66 | **4,64** | 2,14 | | 7 | Col2A1 | 0,24 | 0,50 | 1,24 | **0,66** | 0,30 |
| 14 | | 0,86 | 0,37 | 1,77 | **1,00** | 0,41 | | 14 | | 0,33 | 3,47 | 0,36 | **1,39** | 1,04 |
| 21 | | 1,62 | 3,12 | 2,17 | **2,30** | 0,44 | | 21 | | 0,93 | 2,61 | 0,95 | **1,49** | 0,56 |

**Table S3.** Fold change in mRNA expression for adipogenesis related genes (*CEBPα. PPARγ*) in DPSCs and UC-MSCs by Real-Time RT-PCR. Grey color indicates value of change of fold change. The darker shades of gray correspond to a higher fold change in expression for tested gene.

| **DPSCs** | | Mean fold change | | | **Mean from 3 experiments** | SEM |
|---|---|---|---|---|---|---|
| Day | Gene | Sample 1 | Sample 2 | Sample 3 | | |
| 7 | PPARγ | 15,98 | 3,80 | 2,01 | **7,26** | 4,39 |
| 14 | | 29,05 | 10,81 | 3,20 | **14,35** | 7,67 |
| 21 | | 34,30 | 10,66 | 2,61 | **15,86** | 9,51 |
| 7 | CEBPα | 30,99 | 0,54 | 3,00 | **11,51** | 9,77 |
| 14 | | 3,35 | 0,65 | 3,75 | **2,58** | 0,97 |
| 21 | | 7,20 | 1,02 | 10,74 | **6,32** | 2,84 |

| **UC-MSCs** | | Mean fold change | | | **Mean from 3 experiments** | SEM |
|---|---|---|---|---|---|---|
| Day | Gene | Sample 1 | Sample 2 | Sample 3 | | |
| 7,00 | PPARγ | 16,52 | 11,14 | 17,04 | **14,90** | 1,88 |
| 14,00 | | 15,28 | 23,11 | 32,72 | **23,70** | 5,04 |
| 21,00 | | 80,85 | 68,38 | 67,08 | **72,11** | 4,39 |
| 7,00 | CEBPα | 1,21 | 1,68 | 1,81 | **1,57** | 0,18 |
| 14,00 | | 6,81 | 8,05 | 5,53 | **6,80** | 0,73 |
| 21,00 | | 52,02 | 22,38 | 19,43 | **31,28** | 10,41 |

**Table S4.** Fold change in mRNA expression for cardiomyogenesis related genes (*Gata-4. Nkx2.5. Myl2c*) in DPSCs and UC-MSCs by Real-Time RT-PCR. Grey color indicates value of change of fold change. The darker shades of gray correspond to a higher fold change in expression for tested gene.

| **DPSCs** | | Mean fold change | | | **Mean from 3 experiments** | SEM |
|---|---|---|---|---|---|---|
| Day | Gene | Sample 1 | Sample 2 | Sample 3 | | |
| 7 | Gata-4 | 0,13 | 1,29 | 1,32 | **0,91** | 0,39 |
| 14 | | 1,45 | 0,05 | 0,53 | **0,68** | 0,41 |
| 21 | | 0,26 | 0,09 | 0,16 | **0,17** | 0,05 |
| 7 | Nkx2.5 | 2,79 | 0,60 | 0,69 | **1,36** | 0,71 |
| 14 | | 1,26 | 0,10 | 0,19 | **0,52** | 0,37 |
| 21 | | 0,39 | 0,08 | 0,56 | **0,34** | 0,14 |
| 7 | Myl2c | 1,95 | 1,05 | 0,23 | **1,08** | 0,50 |
| 14 | | 1,12 | 0,02 | 0,81 | **0,65** | 0,33 |
| 21 | | 1,10 | 1,30 | 0,13 | **0,84** | 0,36 |

| **UC-MSCs** | | Mean fold change | | | **Mean from 3 experiments** | SEM |
|---|---|---|---|---|---|---|
| Day | Gene | Sample 1 | Sample 2 | Sample 3 | | |
| 7 | Gata-4 | 6,11 | 7,96 | 0,92 | **5,00** | 2,11 |
| 14 | | 0,48 | 0,04 | 0,46 | **0,33** | 0,14 |
| 21 | | 10,10 | 0,44 | 1,61 | **4,05** | 3,05 |
| 7 | Nkx2.5 | 7,18 | 103,60 | 0,87 | **37,22** | 33,24 |
| 14 | | 1,18 | 0,17 | 0,94 | **0,76** | 0,30 |
| 21 | | 34,47 | 8,62 | 1,28 | **14,79** | 10,06 |
| 7 | Myl2c | 1,21 | 8,75 | 0,49 | **3,48** | 2,64 |
| 14 | | 0,72 | 0,08 | 0,05 | **0,28** | 0,22 |
| 21 | | 5,04 | 9,82 | 0,34 | **5,07** | 2,74 |

**Table S5.** Fold change in mRNA expression for endothelial related genes (*Gata-2. Tie-2.* VE-cadherin) in DPSCs and UC-MSCs by Real-Time RT-PCR. Grey color indicates value of change of fold change. The darker shades of gray correspond to a higher fold change in expression for tested gene.

| DPSCs | | Mean fold change | | | **Mean from 3** | | | UC-MSCs | | Mean fold change | | | **Mean from 3** | |
|---|---|---|---|---|---|---|---|---|---|---|---|---|---|---|
| Day | Gene | Sample 1 | Sample 2 | Sample 3 | **experiments** | SEM | | Day | Gene | Sample 1 | Sample 2 | Sample 3 | **experiments** | SEM |
| 7 | Gata-2 | 1,82 | 1,08 | 0,23 | **1,04** | 0,46 | | 7 | Gata-2 | 3,31 | 23,18 | 0,33 | **8,94** | 7,17 |
| 14 | | 0,41 | 0,19 | 0,02 | **0,21** | 0,11 | | 14 | | 17,82 | 3,02 | 0,75 | **7,20** | 5,35 |
| 21 | | 0,29 | 0,25 | 0,22 | **0,25** | 0,02 | | 21 | | 2,00 | 5,30 | 0,33 | **2,54** | 1,46 |
| 7 | VE-cadherin | 1,95 | 0,93 | 1,10 | **1,33** | 0,32 | | 7 | VE-cadherin | 12,29 | 203,15 | 1,17 | **72,21** | 65,55 |
| 14 | | 0,88 | 0,04 | 1,11 | **0,68** | 0,33 | | 14 | | 4,39 | 1,53 | 0,87 | **2,26** | 1,08 |
| 21 | | 0,09 | 0,10 | 0,60 | **0,27** | 0,17 | | 21 | | 0,93 | 11,12 | 1,49 | **4,52** | 3,31 |
| 7 | Tie-2 | 0,57 | 1,09 | 1,06 | **0,90** | 0,17 | | 7 | Tie-2 | 1,26 | 16,10 | 0,16 | **5,84** | 5,14 |
| 14 | | 0,06 | 0,01 | 0,97 | **0,35** | 0,31 | | 14 | | 1,37 | 0,70 | 0,15 | **0,74** | 0,35 |
| 21 | | 0,02 | 0,03 | 0,87 | **0,31** | 0,28 | | 21 | | 0,30 | 14,87 | 0,23 | **5,13** | 4,87 |
